# Thermal stress affects proliferation and differentiation of turkey satellite cells through the mTOR/S6K pathway in a growth-dependent manner

**DOI:** 10.1371/journal.pone.0262576

**Published:** 2022-01-13

**Authors:** Jiahui Xu, Gale M. Strasburg, Kent M. Reed, Sandra G. Velleman

**Affiliations:** 1 Department of Animal Sciences, The Ohio State University, Wooster, OH, United States of America; 2 Department of Food Science and Human Nutrition, Michigan State University, East Lansing, MI, United States of America; 3 Department of Veterinary and Biomedical Sciences, University of Minnesota, St. Paul, MN, United States of America; King Faisal Specialist Hospital and Research Center, SAUDI ARABIA

## Abstract

Satellite cells (SCs) are stem cells responsible for post-hatch muscle growth through hypertrophy and in birds are sensitive to thermal stress during the first week after hatch. The mechanistic target of rapamycin (mTOR) signaling pathway, which is highly responsive to thermal stress in differentiating turkey pectoralis major (p. major) muscle SCs, regulates protein synthesis and the activities of SCs through a downstream effector, S6 kinase (S6K). The objectives of this study were: 1) to determine the effect of heat (43°C) and cold (33°C) stress on activity of the mTOR/S6K pathway in SCs isolated from the p. major muscle of one-week-old faster-growing modern commercial (NC) turkeys compared to those from slower-growing Randombred Control Line 2 (RBC2) turkeys, and 2) to assess the effect of *mTOR* knockdown on the proliferation, differentiation, and expression of myogenic regulatory factors of the SCs. Heat stress increased phosphorylation of both mTOR and S6K in both turkey lines, with greater increases observed in the RBC2 line. With cold stress, greater reductions in mTOR and S6K phosphorylation were observed in the NC line. Early knockdown of *mTOR* decreased proliferation, differentiation, and expression of myoblast determination protein 1 and myogenin in both lines independent of temperature, with the RBC2 line showing greater reductions in proliferation and differentiation than the NC line at 38° and 43°C. Proliferating SCs are more dependent on mTOR/S6K-mediated regulation than differentiating SCs. Thus, thermal stress can affect breast muscle hypertrophic potential by changing satellite cell proliferation and differentiation, in part, through the mTOR/S6K pathway in a growth-dependent manner. These changes may result in irreversible effects on the development and growth of the turkey p. major muscle.

## 1. Introduction

Birds are homeotherms with a limited ability to maintain body temperature [1, 2]. Hatchlings, in particular, have difficulty regulating body temperature as their thermal regulatory systems are poorly developed [3–5]. Poults may encounter either heat or cold thermal stress immediately after hatch when they are shipped, often hundreds of miles, from hatcheries to grower facilities [6]. Thermal stress immediately after hatch can have long-term effects on the growth and structure of pectoralis major (p. major, breast) muscle, including changes in myofiber diameter and spacing for support systems [7–9]. The probability of exposure of hatchlings to thermal stress will likely increase according to climate models that predict increasing frequency of temperature extremes, thus, potentially affecting muscle development and growth. In the chronology of muscle development, the total number of adult myofibers is fully established by the time of hatch [10]. Post-hatch muscle growth proceeds by hypertrophy of the existing fibers, a process mediated by satellite cells (SCs), which are adult stem cells located at the periphery of myofibers [11]. Post-hatch myofiber hypertrophy is driven by accretion of satellite cell (SC) nuclei to existing myofibers [12, 13]. The mitotic activity of poultry SCs is timing-dependent, and peaks during the first week after hatch [14, 15]. Satellite cell activities including proliferation and differentiation are highly responsive to environmental temperatures during this period [16, 17]. Furthermore, proliferating turkey p. major muscle SCs are more responsive to thermal stress than differentiating SCs [16]. Thus, post-hatch thermal challenge may affect muscle development and growth by altering SC activity.

Modern meat-type turkey lines (heavy-weight and fast-growing) have been genetically selected for improved growth rate and breast muscling [17], but structural defects in muscle have increased with the selection for these characteristics [18, 19]. With a higher growth rate, faster-growing birds produce more metabolic heat and have reduced capacity for heat dissipation due to reduced capillary density throughout the p. major muscle compared to slower-growing birds [20, 21], further amplifying effects of thermal stress. Furthermore, formation of excessive hypertrophic myofibers, giant myofibers, in the p. major muscle of faster-growing turkeys reduces the tissue spacing for support systems like connective tissue and capillaries, increasing the incidence of muscle degeneration [18, 19]. Giant myofibers have been hypothesized to arise from increased proliferation and differentiation of p. major muscle SCs [16, 22, 23], and thermal stress may further affect SC hypertrophy [16, 22].

Using transcriptome profiling of SCs by RNA sequencing, Reed et al. [24, 25] showed both thermal stress and selection for increased 16-week bodyweight significantly altered the expression of genes associated with p. major muscle growth and development during SC proliferation and differentiation. Mechanistic target of rapamycin (mTOR) and p70 ribosomal S6 kinase (p70S6K or S6K) signal transduction pathways were among the top five pathways affected by thermal stress during SC differentiation [25]. Furthermore, both proliferation and myogenic differentiation of SCs are regulated by mTOR signal transduction in mice [26–28], pigs [29, 30] and quail [31]. Thus, the mTOR/S6K signaling pathway may be involved in thermal stress-induced changes in the proliferation and differentiation of turkey p major muscle SCs.

Muscle hypertrophy is widely hypothesized to arise from mTOR-mediated protein synthesis [32–34], and mTOR is involved in hypertrophic growth of skeletal muscle in humans [35], rats [36, 37], mice [32, 38, 39], and chickens [33]. The mTOR protein kinase functions in two independent multiprotein complexes: mTOR protein complex 1 and 2 (mTORC1 and mTORC2) [40–42]. As a nutrient sensor [43–45], mTORC1 regulates intracellular protein turnover [46, 47]. Activation of mTORC1 by phosphorylation is typically stimulated by extracellular growth factors through phosphoinositide 3 kinase via protein kinase B (PI3K/Akt) pathway [48, 49]. Although mTORC2 is not as sensitive to nutrients as mTORC1 does, it can also be activated by growth factors in the presence of PI3K [50]. Activated mTORC2 can indirectly activate mTORC1 by phosphorylation of Akt [51, 52]. In skeletal muscle, mTORC1 promotes protein synthesis through its downstream effector protein, S6K [53]. Since the amount of intracellular protein directly determines the size of myofibers, the mTOR/S6K pathway plays an essential role in regulating the hypertrophic growth of skeletal muscle [36, 54–57].

Activity of the mTOR pathway in skeletal muscle is also affected by thermal stress [33, 58, 59]. For example, Ma et al. [33] showed chronic heat stress from day 28 to 42 after hatch decreased chicken breast muscle hypertrophy by suppressing mTOR/S6K signal transduction. In contrast, increased mTOR activity was observed in chicken leg muscle when newly hatched chickens were challenged with chronic cold stress during the first week after hatch [59]. Since changing the activity of mTOR signal transduction affects the proliferation and myogenic differentiation of mammalian [26–30] and avian [31] SCs, mTOR/S6K signal transduction is likely to affect SC-mediated muscle growth during thermal stress in turkey.

Although previous studies have independently shown that thermal stress can affect both mTOR signal transduction [25, 33, 59] and SC proliferation and differentiation [16, 17, 22, 60] in poultry skeletal muscle, few studies have determined if thermal stress-induced changes in SC activity are regulated by the mTOR pathway in a growth-dependent manner. Furthermore, since SCs exhibit their peak mitotic activity [14, 15] and temperature sensitivity [16, 17] during the first week after hatch, thermal stress during this period may affect SC-mediated muscle growth through mTOR signal transduction. Thus, the objectives of this study were: 1) to determine the effect of heat and cold stress on the activity of mTOR/S6K pathway in p. major muscle SCs isolated from one-week-old modern commercial (NC) turkeys compared to those from one-week-old Randombred Control Line 2 (RBC2) turkeys, and 2) to assess the effect of *mTOR* knockdown with small interfering RNA (siRNA) on the proliferation, differentiation, and expression of myogenic regulatory factors of the SCs. The NC turkeys are a heavy, fast-growing meat-type commercial turkey selected for many growth traits including increased body weight and breast muscle yield, whereas the RBC2 turkeys are slower-growing turkeys representing commercial turkeys during the 1960s [61]. The myogenic regulatory factors measured in the current study include myoblast determination factor 1 (*MyoD*) and myogenin (*MyoG*). The expression of *MyoD* is required for proliferation [62] and *MyoG* for differentiation [63, 64]. This study is the first to establish an association among thermal stress, growth selection, mTOR/S6K signal transduction, and SC proliferation and differentiation. Thus, the results provide cellular mechanistic insight regarding signal transduction in the regulation of thermal stress-induced changes in p. major SC mediation of muscle growth and development.

## 2. Materials and methods

### 2.1. Pectoralis major muscle SCs

Satellite cells used in this study were previously isolated from the p. major muscle of one-week-old RBC2 turkeys and one-week-old NC turkeys according to the method of Velleman et al. [23] and were stored in liquid nitrogen until use. To avoid sex effects [23], only SCs isolated from male turkeys were used in this study.

### 2.2. Cell culture for western blot analysis

Approximately 15,000 cells/well were plated in 24-well plates (Greiner Bio-One, Monroe, NC, USA) in Dulbecco’s Modified Eagle’s Medium (DMEM, Sigma-Aldrich, St. Louis, MO, USA) supplemented with 10% chicken serum (Gemini Bio-Products, West Sacramento, CA, USA), 5% horse serum (Gemini Bio-Products), 1% antibiotics-antimycotics (Gemini Bio-Products), and 0.1% gentamicin (Gemini Bio-Products). The plated cells were allowed to attach for 24 h while incubated in a 95% air / 5% CO_2_ incubator (Thermo Fisher Scientific, Waltham, MA, USA) at 38°C. After 24 h of attachment, the plating medium was replaced with McCoy’s 5A growth medium (Sigma-Aldrich) containing 10% chicken serum (Gemini Bio-Products), 5% horse serum (Gemini Bio-Products), 1% antibiotics-antimycotics (Gemini Bio-Products), and 0.1% gentamicin (Gemini Bio-Products). Satellite cells from both lines were randomly assigned to proliferate at 38°C (control), 43°C (heat stress), or 33°C (cold stress) for 72 h, and the growth medium was changed every 24 h. After 72 h of proliferation, the growth medium was replaced with a DMEM differentiation medium containing 3% horse serum, 1% antibiotics-antimycotics, 0.1% gentamicin, 0.1% gelatin, and 1 mg/mL bovine serum albumin (BSA, Sigma-Aldrich). The incubation temperature for each group of cells during the 72 h of differentiation was the same as that during proliferation, and the differentiation medium was changed every 24 h. At 24-hour intervals (72 h of proliferation and 24, 48, and 72 h of differentiation), one plate was removed, rinsed with phosphate buffered saline (PBS, 137 mM NaCl, 2.68 mM KCl, 1.47 mM KH_2_PO_4_, and 7.81 mM Na_2_HPO_4_, pH 7.08) and protein was extracted.

Total protein in each treatment group (38°, 43°, or 33°C) was extracted with a protein extraction buffer [50 mM Tris–HCl, 1% Nonidet P-40, 0.5% sodium deoxycholate, 0.1% sodium dodecyl sulphate (SDS), 150 mM NaCl, 1 mM EDTA, 1 mM Na_3_VO_4_, and protease and phosphatase inhibitors (Thermo Fisher Scientific)]. In brief, 100 μL of ice-cold protein extraction buffer was added to each well of the cell culture plate, and the plate incubated on ice for 15 min. Cells were scraped from each well, and the lysate from each group was incubated in a 1.5 mL micro centrifuge tube for 15 min on ice. A syringe with a 26 G needle was used to aspirate the cell lysate. Each tube was centrifuged at 10,000 rpm for 15 min at 4°C, and the supernatant of each sample was collected. The protein concentration of each sample was measured using the Bradford method [65].

After adjusting samples to the same concentration, each protein lysate was mixed with a denaturation buffer containing 0.1 M tris-HCl, 10% glycerol, 1% β-mercaptoethanol, 0.1% bromophenol blue, and 1% sodium dodecyl sulfate (SDS) was boiled for 10 min to prepare the samples for sodium dodecyl sulfate-polyacrylamide gel electrophoresis (SDS-PAGE). Denatured samples (30 μg/well) and a pre-stained protein standard for molecular weight (Thermal Fisher Scientific) were separated in a 4–12% SDS-PAGE at a constant current of 40 mA in running buffer (25 mM Tris, 200 mM glycine, and 10% SDS, pH 8.3) according to the method of Laemmli [66]. The separated proteins were transferred from the SDS-PAGE to a polyvinylidene difluoride (PVDF) membrane at 200 mA in a 25 mM Tris, 192 mM glycine, and 20% methanol transfer buffer for 150 min on ice. The PVDF membrane was incubated in a blocking buffer (5% non-fat milk, 20% Tween-20, 20 mM Tris-HCl, 500 mM NaCl, pH 7.5) for 1 h at room temperature. After blocking, the PVDF membrane was incubated with a primary antibody diluted in blocking buffer at 4°C overnight. Rabbit anti-mTOR (1:1000 dilution, Cell Signaling Technology, Danvers, MA, USA), rabbit anti-phospho-mTOR at Ser2448 (1:1000 dilution, Cell Signaling Technology), rabbit anti-S6K (1:1000 dilution, Cell Signaling Technology), rabbit anti-phospho-S6K at Thr229 (1:800 dilution, Abcam, Waltham, MA, USA), and rabbit anti-β-actin (1:1000 dilution, Cell Signaling Technology) were used as primary antibodies. The PVDF membrane was gently agitated in washing buffer (blocking buffer without non-fat milk) for 10 min on a platform shaker for three times, and then incubated in a horseradish peroxidase-conjugated goat anti-rabbit secondary antibody (1:1000 dilution, Cell Signaling Technology) in blocking buffer for 2 h at room temperature. After gently agitating the PVDF membrane with the washing buffer on the platform shaker for 10 min for three times, a chemiluminescent substrate (Thermo Fishier Scientific) for digital imaging of the western blot was used to visualize the target protein bands according to the manufacturer’s recommended procedure on a Bio-Rad ChemiDoc XRS (Bio-Rad, Hercules, CA, USA) imaging system. The membrane was then incubated in a restore western blot stripping buffer (Thermal Fishier Scientific) according to the manufacturer’s protocol, prior to incubation with other primary antibodies as described above.

The band density of β-actin was used to normalize the band density of each target protein. The ratio of phosphorylated protein to total protein was calculated from the normalized band densities as described by Zhang et al. [67] to determine the activity of each protein. To ascertain the fold change, the calculated ratio of each treatment group was divided by the calculated ratio of the control group. Thus, the final adjusted ratio of the control group was 1, and the final adjusted ratio of each treatment group represented a fold change compared to the control group. The western blot analysis was repeated using two independent cultures per treatment group per cell line.

### 2.3. Small interfering RNA and SC culture for transfection

Small interfering RNA targeting *mTOR* (Gene bank ID: XM_010723001.3) was designed using Invitrogen Block-iT software (https://rnaidesigner.thermofisher.com/raniexpress/). The siRNA for *mTOR* was a synthesized stealth siRNA duplex (Thermo Fisher Scientific) with the following sequence: sense strand: 5’-CAA AGA UGA CUG GUU GGA AUG GUU A-3’; anti-sense strand: 5’-UUA CCA UUC CAA GUC AUC UUU G-3’ targeting the *mTOR* open reading frame from 3933 to 3957. A stealth RNAi with 48% GC content (Thermo Fisher Scientific) was used as the negative control siRNA.

To determine the knockdown efficiency of the synthesized *mTOR* siRNA, approximately 18,000 of p. major muscle SCs from both the RBC2 and NC lines were plated per well in 24-well gelatin-coated plates in 500 μl of transfection medium (the plating medium without antibiotics-antimycotics and gentamicin), and incubated in a 95% air / 5% CO2 incubator at 38°C. After 24 h of attachment, cells were transfected with 20 pmol/μl of the negative control siRNA or the *mTOR* siRNA with 1 μl Lipofectamine 2000 (Thermo Fisher Scientific) per well according to the manufacturer’s protocol. After 12 h of transfection, the medium was replaced with growth medium for 72 h of proliferation, and the medium was changed every 24 h. At 72 h post transfection, cells were removed from the incubator and total RNA was extracted for gene expression analysis by Real-Time Quantitative PCR (RT-qPCR) as described in section 2.7. The transfection experiment was repeated twice independently to confirm knockdown efficiency of the *mTOR* siRNA.

For western blot analysis, SCs (~18,000 cells/well) from both lines were plated in 24-well gelatin-coated plates in 500 μl of transfection medium and allowed to attach for 24 h at 38°C. After 24 h, cells were transfected with 20 pmol/μl of the negative control siRNA or the *mTOR* siRNA with 1 μl Lipofectamine 2000 per well. After 12 h of transfection at 38°C, the transfection medium was replaced with growth medium, and SCs from both lines were randomly assigned to a 38°, 43°, or 33°C incubator for 72 h of proliferation. The growth medium was changed every 24 h. After 72 h of proliferation, the growth medium was replaced with differentiation medium, allowed to differentiate for 72 h, with medium replaced every 24 h. Total protein for each treatment group was extracted at 48 h of differentiation as described in section 2.2. Western blot analysis was conducted as described in section 2.2.

For SCs transfected for proliferation analysis, cell culture and transfection methods were the same as the methods of transfection for western blot analysis as described above until 72 h of proliferation. Every 24 h, one plate from each treatment group was removed at 0, 24, 48, and 72 h of proliferation, rinsed with PBS and stored at -70°C for the proliferation assay as described in section 2.4.

For differentiation analysis and measurement of myotube diameter, *mTOR* was either knocked down either at the beginning of proliferation or differentiation.

For knockdown at the beginning of proliferation, cells (~11,000/well) were plated in 48-well gelatin-coated plates in 500 μl of transfection medium and allowed to attach for 24 h in a 95% air / 5% CO_2_ incubator at 38°C. After 24 h, cells were transfected with 20 pmol/μl of the negative control siRNA or the *mTOR* siRNA with 0.5 μl Lipofectamine 2000 per well. After 12 h of transfection at 38°C, the transfection medium was replaced with growth medium, and SCs from both lines were randomly assigned to a 38°, 43°, or 33°C incubator for 72 h. The growth medium was changed every 24 h. After 72 h of proliferation, the growth medium was replaced with differentiation medium and the cells allowed to differentiate for 72 h with the media replaced every 24 h. At 0, 24, 48, and 72 h of differentiation, one plate from each treatment group was removed, rinsed with PBS, and stored at -70°C until analysis for differentiation as described in section 2.5. Ten photomicrographs were randomly taken per treatment group per cell line with an Olympus IX70 (Olympus America, Center Valley, PA, USA) fluorescence microscope at each sampling time for myotube measurement as described in section 2.6.

For *mTOR* knockdown at the beginning of differentiation, SCs (~9,000 cells per well) were plated in 48-well gelatin-coated plates in plating medium and allowed to attach for 24 h at 38°C. After 24 h, plating medium was replaced with growth medium, and the cells from both lines were randomly assigned to a 38°, 43°, or 33°C incubator for 72 h of proliferation. The growth medium was changed every 24 h. At 72 h of proliferation, the cells were transfected with 20 pmol/μl of the negative control siRNA or the *mTOR* siRNA with 0.5 μl Lipofectamine 2000 per well. After 12 h of transfection at 38°C, the growth medium was replaced with differentiation medium for 72 h of differentiation at the same incubation temperature for each group of cells used during proliferation. Differentiation medium was changed every 24 h, and at 0, 24, 48, and 72 h of differentiation one plate from each treatment group was removed, rinsed with PBS and stored at -70°C for the differentiation assay. Ten photomicrographs were randomly taken per treatment group per cell line with the fluorescence microscope at each sampling time for myotube measurement as described in section 2.6.

Cell culture and transfection methods for gene expression analysis were the same as those used for western blot analysis described above. One plate from each treatment group was removed to -70°C at 72 h of proliferation and 48 h of differentiation until RNA extraction. The RT-qPCR was conducted as described in section 2.7.

### 2.4. Proliferation assay

Cell proliferation was measured according to the method of McFarland et al. [68]. All plates were removed from -70°C and thawed at room temperature for 15 min, 200 μl of 0.05% trypsin-EDTA (Thermo Fisher Scientific) in 10 mM Tris, 2 M NaCl, and 1 mM EDTA (TNE) was added to each culture well and incubated at room temperature for 7 min. The plates were then returned to -70°C overnight. After thawing the plates for 15 min at room temperature, 1.8 mL of TNE buffer containing 0.2% (1 mg/ml) Hoechst dye (Sigma-Aldrich) was added to each well, and the plates were gently agitated for 2 h at room temperature. DNA-incorporated Hoechst dye was measured using a Fluoroskan Ascent FL plate reader (Thermo Fisher Scientific). A standard curve with double-stranded calf thymus DNA (Sigma-Aldrich) was used to determine sample DNA concentration. The proliferation assay was repeated in two independent cultures with 4 wells per treatment group per cell line.

### 2.5. Differentiation assay

Satellite cell differentiation was determined by measuring creatine kinase activity using a modified method of Yun et al. [69]. All plates were removed from -70°C and thawed at room temperature for 15 min, and 500 μl of creatine kinase buffer [20 mM glucose (Thermo Fisher Scientific), 20 mM phosphocreatine (Calbiochem, San Diego, CA, USA), 10 mM mg acetate (Thermo Fisher Scientific), 10 mM adenosine monophosphate (Sigma-Aldrich), 1 mM adenosine diphosphate (Sigma-Aldrich), 1 Unit(U)/ml glucose-6-phosphate dehydrogenase (Worthington Biochemical, Lakewood, NJ, USA), 0.5 U/mL hexokinase (Worthington Biochemical), 0.4 mM thio-nicotinamide adenine dinucleotide (Oriental Yeast Co., Tokyo, Japan), 1 mg/mL BSA, to 0.1 M glycylglycine, pH 7.5] was added to each well including the standard curve wells containing creatine phosphokinase with concentrations from 0 to 140 milliunits/well (mU/well, Sigma-Aldrich). The optical density of each well was measured at a wavelength of 405 nm using a BioTek ELx800 (BioTek, Winooski, VT, USA) plate reader. The differentiation assay was repeated in two independent cultures with 5 wells per treatment group per cell line.

### 2.6. Myotube diameter measurement

Photomicrographs were taken with an Olympus IX70 fluorescence microscope equipped with a QImaging Retiga Exi Fast digital camera (Qimaging, Surrey, British Columbia, Canada) and CellSens software (Olympus America) at 48 h of differentiation. The diameter of the myotubes was measured using Image Pro Software (Media Cybernectics, Rockville, MD, USA). One hundred measurements were taken per treatment group per cell line with measurements repeated in two independent cultures with 5 replicate wells per treatment group per cell line.

### 2.7. Gene expression analysis

Extraction of total RNA from each sample was conducted using RNAzol (Molecular Research Center, Cincinnati, OH, USA) based on manufacturer’s procedures. The concentration of each RNA sample was quantified with a spectrophotometer (NanoDrop^TM^ ND-1000, Thermo Fisher Scientific). Reverse transcription was performed using Moloney Murine Leukemia Virus Reverse Transcriptase (M-MLV; Promega, Madison, WI, USA) to produce cDNA from total RNA. The RT-qPCR was done using a DyNAmo Hot Start SYBR Green qPCR kit (Thermo Fisher Scientific) to quantify the expression of *MyoD*, *MyoG*, *mTOR*, and a normalizer gene glyceraldehyde-3-phosphate dehydrogenase (*GAPDH)*. Primers for *MyoD*, *MyoG*, and *GAPDH* (Table 1) were previously designed, and the specificity confirmed as reported in Clark et al. [22]. Primers for *mTOR* were designed using primer-BLAST tool (https://www.ncbi.nlm.nih.gov/tools/primer-blast/), and the specificity of the *mTOR* primers was confirmed by DNA sequencing the PCR product (Molecular and Cellular Imaging Center, The Ohio State University, Wooster, OH, USA). The RT-qPCR reaction was run in a DNA Engine Opticon 2 real-time machine (Bio-Rad) and included: denaturation for 30s at 94°C; annealing for 30 s at 58°C for *MyoD* and *MyoG* or at 55°C for *GAPDH and mTOR*; and elongation for 30 s at 72°C; and a final elongation at 72°C for 5 min in a DNA Engine Opticon 2 real-time machine (Bio-Rad). A standard curve of each gene was generated using serial dilutions of purified PCR products [70]. Arbitrary concentrations from 1 to 100,000 were assigned to each serial dilution. Since the concentration of each amplified cDNA sample was within the concentration range of the corresponding standard curve, the arbitrary molar concentration of each amplified sample was calculated according to the threshold cycle. The arbitrary molar concentration of each cDNA sample was normalized using *GAPDH*. The RT-qPCR for each gene was repeated in two independent cultures with 12 replicate wells per treatment group per cell line.

**Table 1:** Primer sequences for real-time quantitative polymerase chain reaction.

Primer	Sequence	Product size	GenBank accession number
*MyoD* ^1^	5’-GAC GGC ATG ATG GAG TAC AG-3’ (forward)	201 bp^5^	AY641567.1
	5’-AGC TTC AGC TGG AGG CAG TA-3’ (reverse)		
*MyoG* ^2^	5’-CCT TTC CCA CTC CTC TCC AAA-3’ (forward)	175 bp	AY560111.3
	5’-GAC CTT GGT CGA AGA GCA ACT-3’ (reverse)		
*mTOR* ^3^	5’-GGG TGG AGG TGA ACT TAC AGG-3’ (forward)	185 bp	XM_010723001.3
	5’-TCC TGG CTC ATT TCA CGG AG-3’ (reverse)		
*GAPDH* ^4^	5’-GAG GGT AGT GAA GGC TGC TG-3’ (forward)	200 bp	U94327.1
	5’-CCA CAA CAC GGT TGC TGT AT-3’ (reverse)		

^1^*MYOD*, Myogenic Determination Factor 1

^2^*MYOG*, Myogenin

^3^*mTOR*, Mechanistic Target of Rapamycin

^4^*GAPDG*, Glyceraldehyde-3-phosphate dehydrogenase

^5^bp, number of base pairs

### 2.8. Statistical analysis

Data from the western blot assays were analyzed as a completely randomized design with two treatment factors: temperature (heat or cold stress) and line (RBC2 or NC) at each sampling time. The model included the main effects, an interaction effect of line × temperature, and a random effect of experimental repeat. Mean and standard error of the mean (SEM) for each treatment group was determined by least square means statement in the MIXED procedure in SAS (SAS 9.4, SAS Institute INC., Cary, NC, USA). Differences between each mean value were separated by the pdiff option in SAS and *P* ≤ 0.05 was considered statistically significant.

Data from the *mTOR* knockdown experiments for western blot, proliferation and differentiation assays, myotube measurement, and gene expression analysis were analyzed as a completely randomized design with three treatment factors: temperature (heat or cold stress), knockdown (*mTOR* or negative control), and line (RBC2 or NC) at each sampling time. The model included the main effects of temperature, knockdown, and line, an interaction effect of line × temperature, an interaction effect of line × knockdown, an interaction effect of temperature × knockdown, an interaction effect of line × temperature × knockdown, and a random effect of repeat experiment. Mean and SEM for each treatment group were determined by least square means statement in the MIXED procedure in SAS. Differences between each mean value were separated by the pdiff option in SAS. For the data from proliferation assay and differentiation assay, REG procedure in SAS was used to evaluate the linear regression as a function of sampling time in each treatment group of each cell line at each temperature. Contrast statement was used to determine the difference in linear regression and *P* ≤ 0.05 was considered statistically significant.

## 3. Results

### 3.1. Effect of thermal stress and growth selection on mTOR/S6K phosphorylation

#### 3.1.1 Effect of heat stress

**Fig 1:**
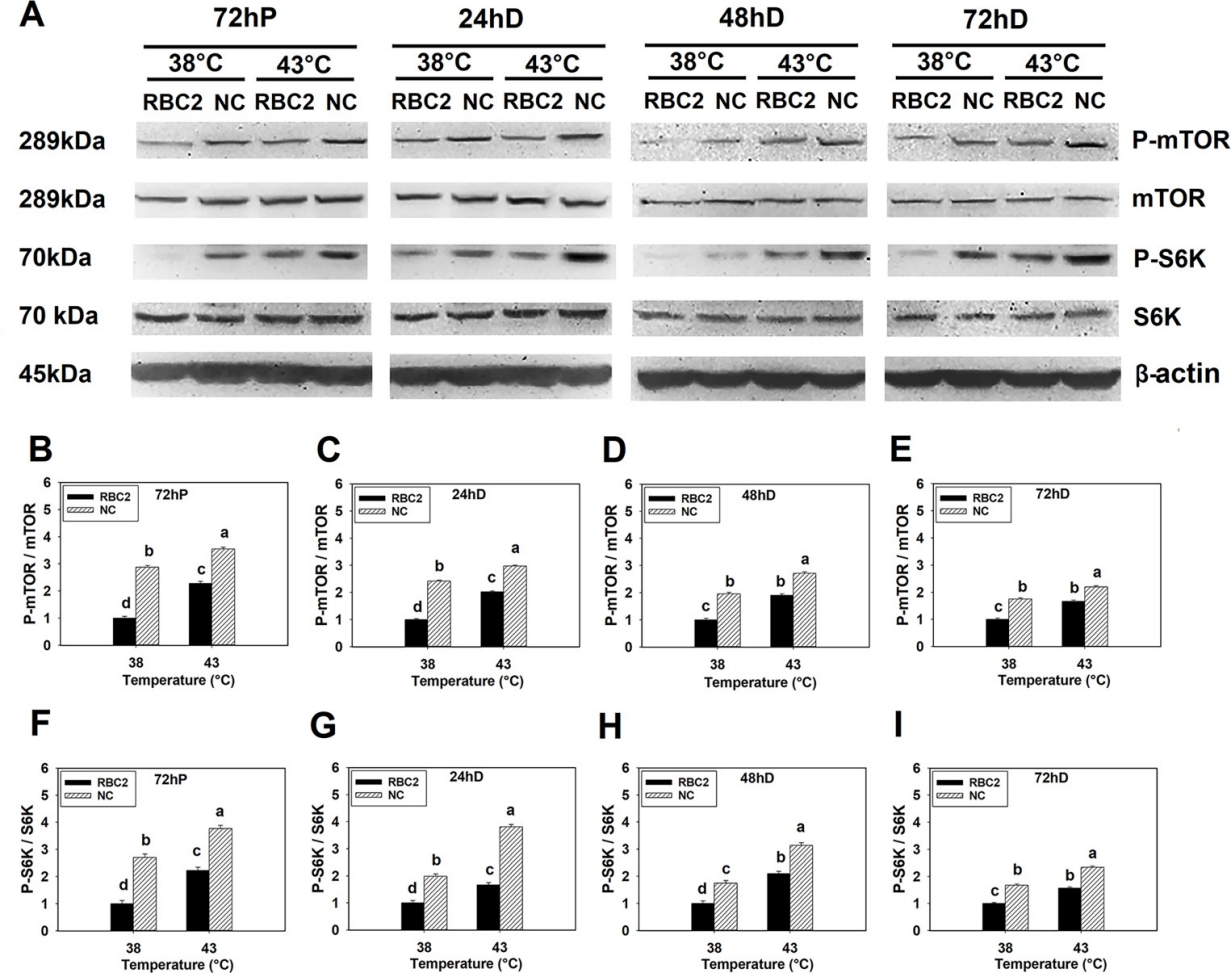
Effect of heat stress on the phosphorylation of mTOR and S6K in RBC2 and NC line SCs. (A) Protein levels of the unphosphorylated and phosphorylated forms of mTOR and S6K, and an internal control β-actin in SCs from the RBC2 and NC lines cultured at 38° or 43°C determined by western blot analysis at 72 h of proliferation (72hP) and 24 h (24hD), 48 (48hD), and 72 h (72hD) of differentiation. Molecular weight and name of each target protein is shown on the left and right side of each figure, respectively. The densitometric ratio of phosphorylated to unphosphorylated mTOR was analyzed in each treatment group at 72hP (B), 24hD (C), 48hD (D), and 72hD (E). The densitometric ratio of phosphorylated to unphosphorylated S6K was analyzed in each treatment group at 72hP (F), 24hD (G), 48hD (H), and 72hD (I). Each graph bar represents a mean ratio, and each error bar represents a standard error of the mean value. Mean values without the same letter are significantly different (*P* ≤ 0.05).

Heat stress (43°C) increased phosphorylation of mTOR in both the RBC2 (*P* < 0.0001) and NC (*P* ≤ 0.0001) line SCs compared to control temperature (38°C) at 72 h of proliferation and 24, 48, 72 h of differentiation (Fig 1A–1E). A greater phosphorylation in mTOR was observed in the NC line SCs compared to the RBC2 line at each sampling time (*P* < 0.0001, Fig 1A–1E). A significant interaction effect was observed between temperature and line at 72 h of proliferation (*P* < 0.0001) and 24 h (*P* = 0.0002), 48 h (*P* = 0.0008), and 72 h (*P* = 0.0006) of differentiation (Fig 1A–1E).

In response to heat stress (43°C), phosphorylation of S6K was increased in both line SCs compared to the control temperature (38°C) at all sampling times (*P* ≤ 0.0006, Fig 1A and 1F–1I). In all comparisons, phosphorylation of S6K was greater in the NC line SCs compared to the RBC2 line (*P* ≤ 0.0008, Fig 1A and 1F–1I). A significant interaction effect between temperature and line occurred at 72 h of proliferation (*P* < 0.0001), and 24 h (*P* = 0.0003), 48 h (*P* = 0.0008), and 72 h (*P* = 0.0009) of differentiation (Fig 1A and 1F–1I).

#### 3.1.2 Effect of cold stress

**Fig 2:**
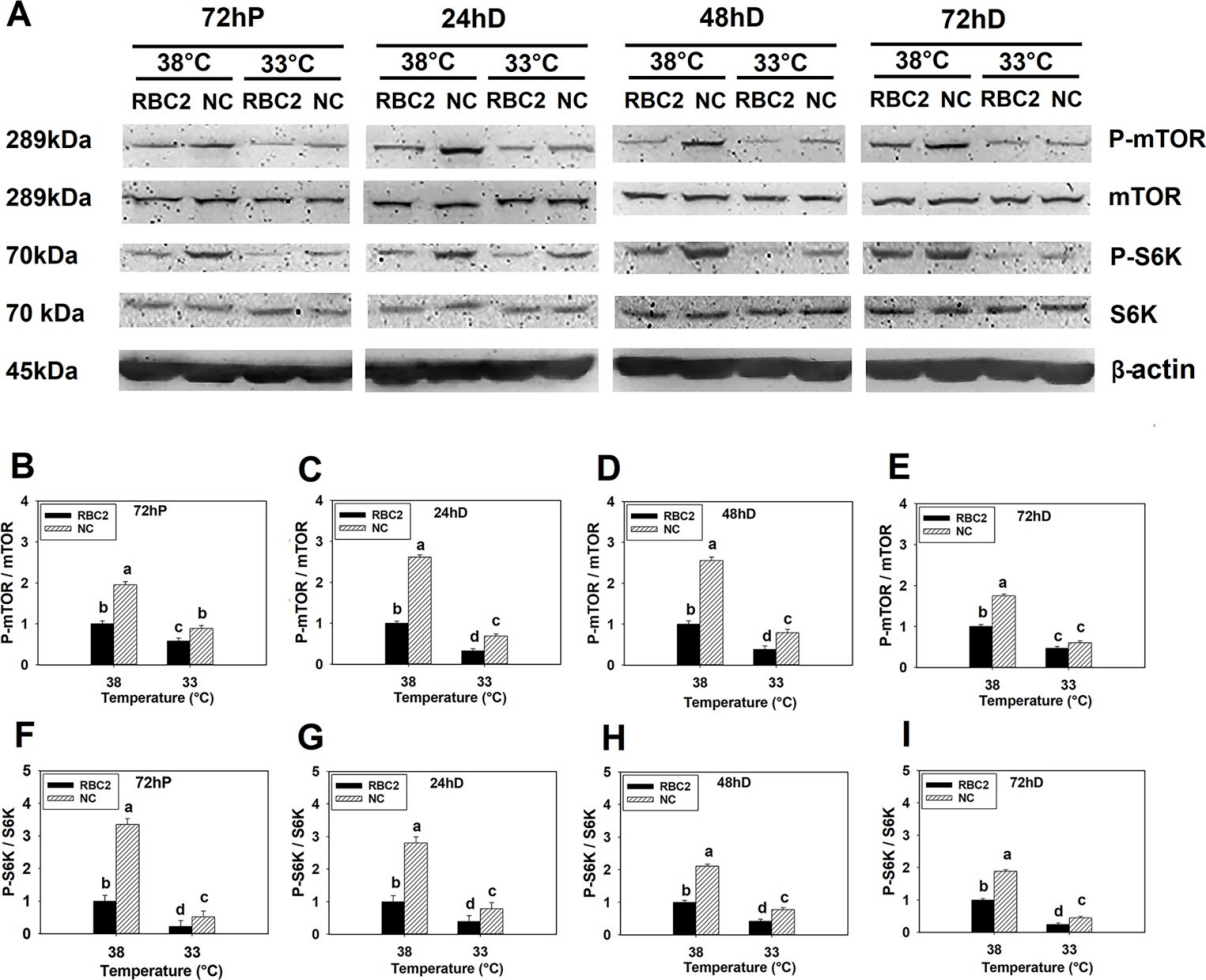
Effect of cold stress on phosphorylation of mTOR and S6K in RBC2 and NC line SCs. (A) Protein levels of the unphosphorylated and phosphorylated forms of mTOR and S6K, and an internal control β-actin in SCs from the RBC2 and NC lines cultured at 38° or 33°C determined by western blot analysis at 72 h of proliferation (72hP) and 24 h (24hD), 48 h (48hD), and 72 h (72hD) of differentiation. Molecular weight and name of each target protein is shown on the left and right side of each figure, respectively. The densitometric ratio of phosphorylated to unphosphorylated mTOR was analyzed in each treatment group at 72hP (B), 24hD (C), 48hD (D), and 72hD (E). The densitometric ratio of phosphorylated to unphosphorylated S6K was analyzed in each treatment group at 72hP (F), 24hD (G), 48hD (H), and 72hD (I). Each graph bar represents a mean ratio, and each error bar represents a standard error of the mean value. Mean values with different letters are significantly different (*P* ≤ 0.05).

In response to cold stress (33°C), phosphorylation of mTOR was lower in both the RBC2 (*P* < 0.0001) and NC (*P* < 0.0001) line SCs than the levels observed at 38°C at all sampling times (Fig 2A–2E). Phosphorylation was higher at 33°C in the NC line SCs compared to the RBC2 line at 72 h of proliferation, 24 and 48 h of differentiation (*P* < 0.0001, Fig 2A–2D), but not significantly different at 72 h of differentiation (*P* = 0.0778, Fig 2A and 2E). There was a significant interaction between line and temperature effects at 72 h of proliferation (*P* < 0.0001) and 24 h (*P* < 0.0001), 48 h (*P* < 0.0001), and 72 h (*P* = 0.0005) of differentiation (Fig 2A–2E).

Phosphorylation of S6K was decreased in SCs of both lines at 33°C compared to the control temperature (38°C) at all sampling times points (*P* < 0.0001, Fig 2A and 2F–2I). Similar to mTOR, phosphorylation of S6K was greater at 33°C in the NC line SCs compared to the RBC2 line at all sampling times (*P* ≤ 0.0007, Fig 2A and 2F–2I). A significant interaction effect was also observed between temperature and line at 72 h of proliferation (*P* < 0.0001) and 24 h (*P* < 0.0001), 48 h (*P* < 0.0001), and 72 h (*P* = 0.0003) of differentiation (Fig 2A and 2F–2I).

### 3.2. Knockdown expression of mTOR in turkey p. major muscle SCs

**Fig 3:**
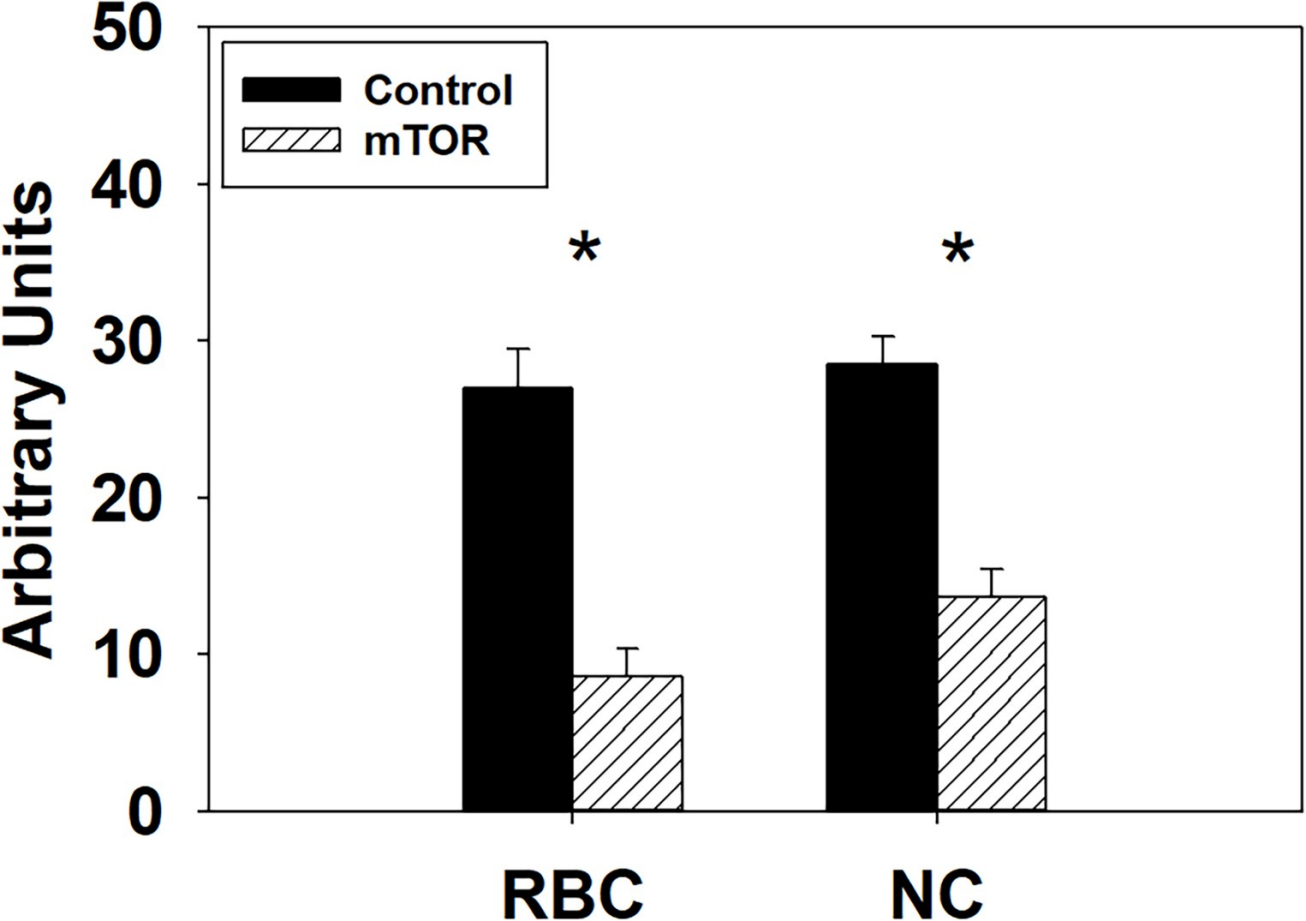
Knockdown efficiency of the *mTOR* siRNA. Relative expression of *mTOR* as determined by RT-qPCR at 72 h of proliferation after knocking down *mTOR* with small interfering RNA at the beginning of proliferation in SCs from the RBC2 and NC turkeys. Asterisk (*) above the bars represents a significant difference between the two adjacent groups (*P* ≤ 0.05).

To confirm the knockdown of *mTOR* mRNA expression by the siRNA, SCs from both lines were transfected with either an *mTOR* siRNA or a negative control siRNA at the beginning of proliferation at the control temperature (38°C) and examined by RT-qPCR. At 72 h post transfection, expression of *mTOR* decreased 3.15-fold (*P* < 0.0001) and 2.08-fold (*P* < 0.0001) in the RBC2 and NC *mTOR* knockdown groups compared to the negative control groups (Fig 3).

### 3.3. Effect of mTOR knockdown, thermal stress, and growth selection on mTOR/S6K phosphorylation

Phosphorylation profiles of both mTOR and S6K (ratio of phosphorylated to unphosphorylated mTOR and S6K) during 72 h of differentiation without knockdown of *mTOR* are presented in S1 Fig. Phosphorylation of both mTOR and S6K linearly increased (*P* < 0.0001) in both the RBC2 and NC line SCs from 72 h of proliferation to 48 h of differentiation at 38°, 43°, and 33°C (S1A–S1I Fig). Furthermore, phosphorylation of mTOR and S6K peaked in both lines at 48 h of differentiation in the heat treatment groups (S1D–S1F Fig). Thus, 48 h of differentiation was chosen as a sampling time for determining the effect of *mTOR* knockdown on the activity of mTOR/S6K pathway.

#### 3.3.1 Effect of heat stress

**Fig 4:**
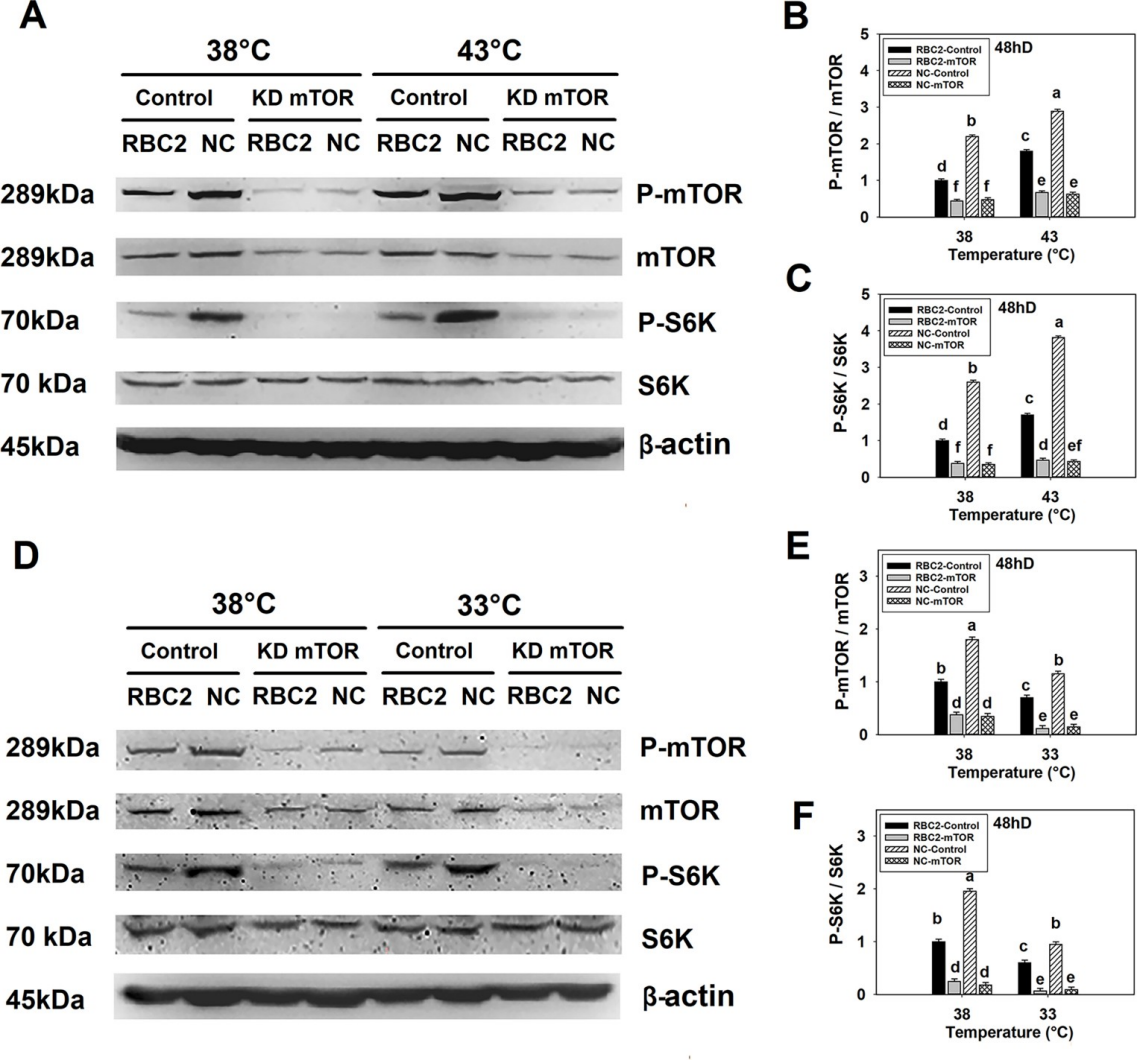
Effects of heat or cold thermal stress and siRNA-mediated *mTOR* knockdown at the beginning of proliferation on the phosphorylation of mTOR and S6K in RBC2 and NC line SCs. (A) Protein levels of the unphosphorylated and phosphorylated forms of mTOR and S6K, and an internal control β-actin in SCs from the RBC2 and NC lines cultured at 38° and 43°C was determined by western blot analysis at 48 h of differentiation. (B) The densitometric ratio of phosphorylated to unphosphorylated mTOR as shown in (A) was analyzed in each treatment group. (C) The densitometric ratio of phosphorylated to unphosphorylated S6K as shown in (A) was analyzed in each treatment group. (D) Protein expression of the unphosphorylated and phosphorylated forms of mTOR and S6K, and the protein expression of an internal control β-actin in SCs from the RBC2 and NC lines cultured at 38° and 33°C was determined by western blot analysis at 48 of differentiation. (E) The densitometric ratio of phosphorylated to unphosphorylated mTOR as shown in (D) was analyzed in each treatment group. (F) The densitometric ratio of phosphorylated to unphosphorylated S6K as shown in (D) was analyzed in each treatment group. Each treatment is shown above each lane in (A) and (D), “Control” group represents cells were transfected with a negative control siRNA, and “KD mTOR” group represents cells were transfected with an *mTOR* targeting siRNA. Molecular weight and name of each target protein is shown on the left and right side of each figure, respectively in (A) and (D). Each graph bar represents a mean ratio, and each error bar represents a standard error of the mean value. Mean values with different letters are significantly different (*P* ≤ 0.05).

When *mTOR* was knocked down at the beginning of proliferation, levels of both the phosphorylated and unphosphorylated forms of mTOR decreased in both lines at 38° and 43°C (*P* < 0.0001, Fig 4A and 4B). At 38°C, phosphorylation of mTOR decreased 2.27-fold (*P* < 0.0001) and 4.58-fold (*P* < 0.0001) in the RBC2 and NC line SCs, respectively, and at 43°C, phosphorylation was reduced 2.69-fold (*P* < 0.0001) and 4.60-fold (*P* < 0.0001) in the RBC2 and NC line SCs, respectively, compared to the control groups (Fig 4A and 4B). A significant interaction effect (*P* < 0.0001) among the effects of temperature, line, and knockdown of *mTOR* was observed at 48 h of differentiation (Fig 4A and 4B).

When *mTOR* was knocked down at the beginning of proliferation, phosphorylation of S6K also significantly decreased at both 38° and 43°C (Fig 4A and 4C). A 2.63-fold (*P* < 0.0001) and 7.63-fold (*P* < 0.0001) reduction was observed in the RBC2 and NC *mTOR* knockdown groups compared to the control groups at 38°C (Fig 4A and 4C). A 3.62-fold (*P* < 0.0001) and 8.87-fold (*P* < 0.0001) reduction was observed in the RBC2 and NC *mTOR* knockdown groups at 43°C compared to the controls (Fig 4A and 4C). A significant interaction effect among the effects of temperature, line, and knockdown of *mTOR* was observed at 48 h of differentiation (*P* < 0.0001, Fig 4A and 4C).

#### 3.3.2 Effect of cold stress

At 38° and 33°C, *mTOR* knockdown at the beginning of proliferation resulted in a significant decrease (*P* < 0.0001) in both the phosphorylated and unphosphorylated form of mTOR expression in SCs from both lines (Fig 4D and 4E). In the *mTOR* knockdown groups, a 5.83-fold (*P* < 0.0001) and 7.69-fold (*P* < 0.0001) reduction was observed in the RBC2 and NC lines compared to the control groups at 33°C (Fig 4D and 4E). There was a significant interaction effect among the effects of temperature, line, and knockdown of *mTOR* at 48 h of differentiation (*P* < 0.0001, Fig 4D and 4E).

At 48 h of differentiation, phosphorylation of S6K was also lower in the RBC2 and NC *mTOR* knockdown groups compared to the control groups at 38° and 33°C (*P* < 0.0001, Fig 4D and 4F). At 33°C, phosphorylation of S6K was decreased 8.57-fold (*P* < 0.0001) and 10.59-fold (*P* < 0.0001) in the RBC2 and NC *mTOR* knockdown groups compared to the controls (Fig 4D and 4F). There was significant interaction (*P* < 0.0001) among the effects of temperature, line, and knockdown of *mTOR* at 48 h of differentiation (Fig 4D and 4F).

### 3.4. Effect of mTOR knockdown, thermal stress, and growth selection on SC proliferation

#### 3.4.1 Effect of heat stress

After knocking down *mTOR* expression at the beginning of proliferation, SCs from both lines were incubated at 38° or 43°C for 72 h of proliferation. Proliferation was measured at 0, 24, 48, and 72 h (Table 2). At 38°C with *mTOR* knocked down, proliferation was 1.98-fold (*P* = 0.0112) and 2.20-fold (*P* < 0.0001) lower in the RBC2 and NC line SCs compared to the control groups only at 72 h. In contrast, at 43°C, SC proliferation was reduced in the RBC2 line in the *mTOR* knockdown group at 72 h (1.23-fold, *P* < 0.0001). In the NC line, proliferation was lower in the *mTOR* knockdown group at 24, 48, and 72 h [a 1.58-fold (*P* = 0.0004), 1.79-fold (*P* < 0.0001), and 2.05-fold (*P* < 0.0001) decrease, respectively]. Proliferation of the SCs in both lines showed a linear increase (*P* < 0.0001) in the control and *mTOR* knockdown groups at 38° and 43°C as a function of time. When *mTOR* was knocked down, the slope of linear regression decreased 3.34-fold (*P* < 0.0001) and 2.55-fold (*P* < 0.0001) in the RBC2 and NC line SCs at 38°C. At 43°C, the slope of the linear regression was 3.41-fold (*P* < 0.0001) and 1.67-fold (*P* < 0.0001) smaller in the RBC2 and NC lines SCs in the *mTOR* knockdown groups compared to the controls. No significant interaction effect was observed among the effects of temperature, line, and knockdown of *mTOR* of proliferation. However, significant interaction effects were observed between temperature and line (*P* = 0.0447), between line and knockdown of *mTOR* (*P* = 0.0002), and between temperature and knockdown of *mTOR* (*P* < 0.0001) at 72 h.

**Table 2:** Effect of heat stress (43°C) and knockdown of *mTOR* on RBC2 and NC line SC proliferation^1^.

Line	Temperature^2^	Knockdown^3^	Sampling time				*P*-value^4^
			0 h	24 h	48 h	72 h	
RBC2	38	Control	0.14^a^^,^^y^ ± 0.01	0.19^cd^^,^^y^ ± 0.03	0.31^de^^,^^y^ ± 0.10	0.62^d^^,^^z^ ± 0.08	< 0.0001
		mTOR	0.13^a^^,^^y^ ± 0.00	0.13^d^^,^^y^ ± 0.03	0.17^e^^,^^y^ ± 0.09	0.31^e^^,^^z^ ± 0.08	< 0.0001
	43	Control	0.13^a^^,^^x^ ± 0.01	0.20^bcd^^,^^x^ ± 0.04	0.51^cd^^,^^y^ ± 0.10	1.57^b^^,^^z^ ± 0.08	< 0.0001
		mTOR	0.13^a^^,^^x^ ± 0.00	0.13^d^^,^^x^ ± 0.03	0.24^de^^,^^y^ ± 0.10	0.71^d^^,^^z^ ± 0.08	< 0.0001
NC	38	Control	0.13^a^^,^^x^ ± 0.01	0.26^bc^^,^^x^ ± 0.03	0.63^bc^^,^^y^ ± 0.10	1.46^bc^^,^^z^ ± 0.08	< 0.0001
		mTOR	0.13^a^^,^^x^ ± 0.00	0.18^cd^^,^^x^ ± 0.03	0.38^cde^^,^^y^ ± 0.10	0.66^d^^,^^z^ ± 0.09	< 0.0001
	43	Control	0.14^a^^,^^w^ ± 0.01	0.46^a^^,^^x^ ± 0.03	1.43^a^^,^^y^ ± 0.10	2.67^a^^,^^z^ ± 0.10	< 0.0001
		mTOR	0.14^a^^,^^x^ ± 0.01	0.29^b^^,^^x^ ± 0.03	0.80^b,^^y^ ± 0.10	1.30^c^^,^^z^ ± 0.11	< 0.0001
*P*-value^5^		L	0.8123	<0.0001	<0.0001	<0.0001	
		T	0.5876	0.0008	<0.0001	<0.0001	
		K	0.4948	0.0002	<0.0001	<0.0001	
		L × T × K	0.7781	0.2904	0.3800	0.9826	

^1^ Each value represents mean DNA concentration (μg / well) ± Standard error of mean (SEM)

^2^ Incubation temperature (°C) during proliferation and differentiation

^3^ Control = transfecting cells with a negative control sequence; mTOR = knockdown of *mTOR*

^4^ Effect of sampling times within each cell line, temperature, and knockdown of *mTOR*

^5^ Effect of line (L), temperature (T), and knockdown of *mTOR* (K), and the interaction effect among line, temperature, and knockdown of *mTOR* (L × T × K) within each sampling time

^a-f^ Mean DNA concentration (μg / well ± SEM) within a column (sampling time) without a common letter are significantly different

^w-z^ Mean DNA concentration (μg / well ± SEM) within a row (temperature and cell line) without a common letter are significantly different

*P* ≤ 0.05 was considered as significant different

#### 3.4.1 Effect of cold stress

After *mTOR* knockdown at the beginning of proliferation, proliferation was measured at 0, 24, 48, and 72 h (Table 3). Knockdown of mTOR showed no significant effect on SC proliferation in the either line SCs at 48 h (RBC2: *P* = 0.1933, NC: *P* = 0.4694) and 72 h (RBC2: *P* = 0.2111, NC: *P* = 0.6286) at 33°C. With *mTOR* knocked down, the slope of the linear regression of SC proliferation had a 1.37-fold (*P* < 0.0001) and 5.20-fold (*P* < 0.0001) reduction in the RBC2 and NC lines at 33°C. Significant interaction occurred among the effects of temperature, line, and knockdown of *mTOR* at 24 h (*P* = 0.0143) and 72 h (*P* = 0.0013).

**Table 3:** Effect of cold stress (33°C) and knockdown of *mTOR* on RBC2 and NC line SC proliferation^1^.

Line	Temperature^2^	Knockdown^3^	Sampling time				*P*-value^4^
			0 h	24 h	48 h	72 h	
RBC2	38	Control	0.11^a^^,^^x^ ± 0.01	0.17^c^^,^^x^ ± 0.01	0.41^b^^,^^y^ ± 0.03	0.93^c^^,^^z^ ± 0.04	< 0.0001
		mTOR	0.11^a^^,^^y^ ± 0.01	0.15^d^^,^^y^ ± 0.01	0.22^c^^,^^y^ ± 0.03	0.63^d^^,^^z^ ± 0.05	< 0.0001
	33	Control	0.11^a^^,^^w^ ± 0.01	0.13^d^^,^^x^ ± 0.01	0.15^cd^^,^^y^ ± 0.15	0.21^ef^^,^^z^ ± 0.05	< 0.0001
		mTOR	0.10^a^^,^^zy^ ± 0.01	0.09^e^^,^^y^ ± 0.01	0.11^d^^,^^zy^ ± 0.11	0.12^f^^,^^z^ ± 0.05	< 0.0001
NC	38	Control	0.11^a^^,^^w^ ± 0.01	0.29^a^^,^^x^ ± 0.01	0.55^a^^,^^y^ ± 0.03	1.82^a^^,^^z^ ± 0.05	< 0.0001
		mTOR	0.11^a^^,^^w^ ± 0.01	0.21^b^^,^^x^ ± 0.01	0.35^b^^,^^y^ ± 0.03	1.10^b^^,^^z^ ± 0.05	< 0.0001
	33	Control	0.11^a^^,^^x^ ± 0.01	0.18^c^^,^^y^ ± 0.01	0.21^c^^,^^y^ ± 0.02	0.32^e^^,^^z^ ± 0.05	< 0.0001
		mTOR	0.10^a^^,^^x^ ± 0.01	0.16^cd^^,^^y^ ± 0.01	0.19^c^^,^^y^ ± 0.02	0.29^e^^,^^z^ ± 0.05	< 0.0001
*P*-value^5^		L	0.5281	<0.0001	<0.0001	<0.0001	
		T	0.1860	<0.0001	<0.0001	<0.0001	
		K	0.9005	<0.0001	<0.0001	<0.0001	
		L × T × K	0.9862	0.0143	0.6462	0.0013	

^1^ Each value represents mean DNA concentration (μg / well) ± Standard error of mean (SEM)

^2^ Incubation temperature (°C) during proliferation and differentiation

^3^ Control = transfecting cells with a negative control sequence; mTOR = knockdown of *mTOR*

^4^ Effect of sampling times within each cell line, temperature, and knockdown of *mTOR*

^5^ Effect of line (L), temperature (T), and knockdown of *mTOR* (K), and the interaction effect among line, temperature, and knockdown of *mTOR* (L × T × K) within each sampling time

^a-f^ Mean DNA concentration (μg / well ± SEM) within a column (sampling time) without a common letter are significantly different

^w-z^ Mean DNA concentration (μg / well ± SEM) within a row (temperature and cell line) without a common letter are significantly different

*P* ≤ 0.05 was considered as significant different

### 3.5. Effect of mTOR knockdown, thermal stress, and growth selection on SC differentiation

#### 3.5.1 Effect of heat stress

After *mTOR* knockdown at the beginning of proliferation, differentiation was measured at 0, 24, 48, and 72 h of differentiation (Table 4). At both 38° and 43°C, SC differentiation as determined by measuring creatine kinase activity was reduced (*P* < 0.0001) in both the RBC2 and NC *mTOR* knockdown groups compared to the control groups at each sampling time. In all the comparisons, the RBC2 line SCs always showed a greater fold change in differentiation compared to the NC line at both 38° and 43°C. From 0 to 48 h, differentiation of the SCs in both lines showed a linear increase in both the control and *mTOR* knockdown groups at both 38° and 43°C. The slope of the linear regression (from 0 to 48 h) was reduced 7.66-fold (*P* < 0.0001) and 3.22-fold (*P* < 0.0001) in the RBC2 and NC *mTOR* knockdown groups at 38°C, compared to the control group, respectively. At 43°C, the slope of the linear regression (from 0 to 48 h) was smaller in the RBC2 and NC *mTOR* knockdown groups [a 4.02-fold (*P* < 0.0001) and 2.21-fold (*P* < 0.0001) reduction, respectively]. Significant interaction was observed among the temperature, line, and knockdown of *mTOR* effects of at 0 h (*P* = 0.0006) and 24 h (*P* < 0.0001).

**Table 4:** Effect of heat stress (43°C) and knockdown of *mTOR* at the beginning of proliferation on RBC2 and NC line SC differentiation^1^.

Line	Temperature^2^	Knockdown^3^	Sampling time				*P*-value^4^
			0 h	24 h	48 h	72 h	
RBC2	38	Control	5.41^c^^,^^w^ ± 0.61	12.47^e^^,^^x^ ± 1.06	49.53^c^^,^^y^ ± 3.35	67.37^b^^,^^z^ ± 1.38	< 0.0001
		mTOR	2.41^d^^,^^x^ ± 0.55	2.27^g^^,^^x^ ± 1.14	8.80^f^^,^^y^ ± 3.35	11.42^f^^,^^z^ ± 1.10	< 0.0001
	43	Control	8.04^b^^,^^x^ ± 0.56	45.97^b^^,^^y^ ± 1.14	68.50^b^^,^^z^ ± 3.74	72.80^a^^,^^z^ ± 1.26	< 0.0001
		mTOR	4.59^c^^,^^x^ ± 0.51	12.42^e^^,^^y^ ± 1.06	20.04^e^^,^^z^ ± 2.83	20.73^e^^,^^z^ ± 1.26	< 0.0001
NC	38	Control	7.13^b^^,^^w^ ± 0.56	34.08^c^^,^^x^ ± 1.14	74.40^b^^,^^z^ ± 3.74	58.36^c^^,^^y^ ± 1.17	< 0.0001
		mTOR	4.47^c^^,^^y^ ± 0.48	8.50^f^^,^^y^ ± 1.06	26.28^e^^,^^z^ ± 3.35	21.99^e^^,^^z^ ± 1.26	< 0.0001
	43	Control	17.35^a^^,^^x^ ± 0.55	62.18^a^^,^^y^ ± 1.15	86.68^a^^,^^z^ ± 3.74	63.46^b^^,^^y^ ± 1.39	< 0.0001
		mTOR	8.58^b^^,^^w^ ± 0.48	29.06^d^^,^^x^ ± 1.14	39.57^d^^,^^z^ ± 3.35	31.44^d^^,^^y^ ± 1.27	< 0.0001
*P*-value^5^		L	< 0.0001	< 0.0001	< 0.0001	0.4178	
		T	< 0.0001	< 0.0001	< 0.0001	< 0.0001	
		K	< 0.0001	< 0.0001	< 0.0001	< 0.0001	
		L × T × K	0.0006	< 0.0001	0.3787	0.8969	

^1^ Each value represents mean creatine kinase activity (Unit / well) ± Standard error of mean (SEM)

^2^ Incubation temperature (°C) during proliferation and differentiation

^3^ Control = transfecting cells with a negative control sequence; mTOR = knockdown of *mTOR*

^4^ Effect of sampling times within each cell line, temperature, and knockdown of *mTOR*

^5^ Effect of line (L), temperature (T), and knockdown of *mTOR* (K), and the interaction effect among line, temperature, and knockdown of *mTOR* (L × T × K) within each sampling time

^a-f^ Mean creatine kinase activity (Unit / well ± SEM) within a column (sampling time) without a common letter are significantly different

^w-z^ Mean creatine kinase activity (Unit / well ± SEM) within a row (temperature and cell line) without a common letter are significantly different

*P* ≤ 0.05 was considered as significant different

**Fig 5:**
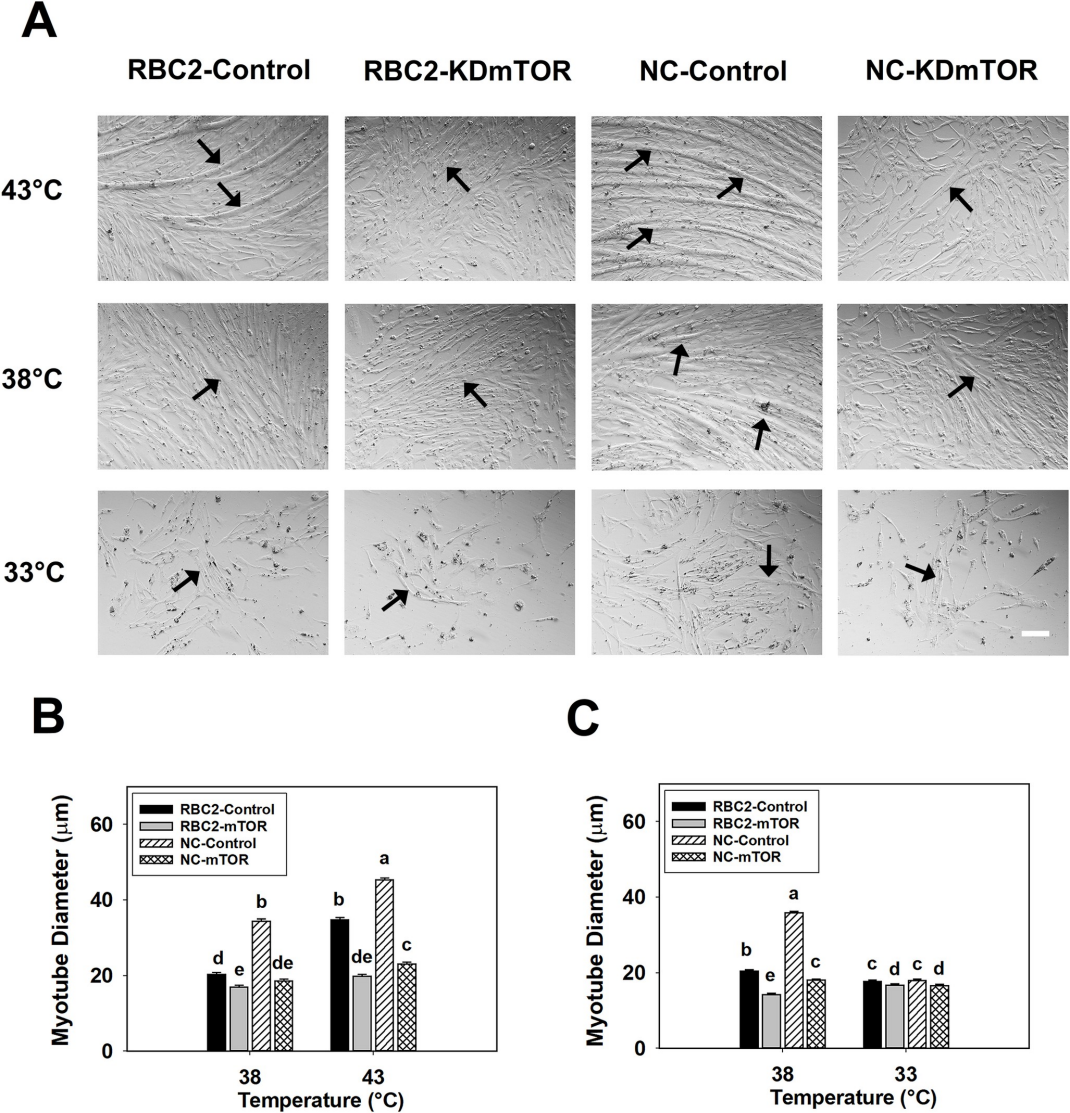
Effect of heat or cold thermal stress and siRNA-mediated knockdown of *mTOR* at the beginning of proliferation on the diameter of myotubes formed by RBC2 and NC line SCs. (A) Representative photomicrographs of SCs from the RBC2 and NC lines transfected with either a negative control siRNA (Control) or a siRNA targeting *mTOR* (KD mTOR) at the beginning of proliferation and followed with 72 h of proliferation and 48 h of differentiation at 38, 43, or 33°C. (B) Diameter of myotubes in the RBC2 and NC lines at 38 or 43°C as shown in (A). (C) Diameter of myotubes in the RBC2 and NC lines at 38 or 33°C as shown in (A). Each graph bar represents a mean diameter of the myotubes, and each error bar represents a standard error of the mean value. Mean values with different letters are significantly different (*P* ≤ 0.05). Black arrows highlight representative myotubes. Scale bar (White) = 100 μm.

Knockdown of mTOR had a significant effect on myotube diameter at 48 h of differentiation at 38°C, where the diameter of myotubes in the RBC2 and NC *mTOR* knockdown groups decreased 1.20-fold (*P* < 0.0001) and 1.86-fold (*P* < 0.0001) compared to the controls (Fig 5A and 5B). At 43°C, myotube diameter in the RBC2 and NC knockdown groups was 1.76-fold (*P* < 0.0001) and 1.96-fold (*P* < 0.0001) smaller at 48 h of differentiation (Fig 5A and 5B). Significant interaction was observed among the effects of temperature, line, and knockdown of *mTOR* at 48 h of differentiation (*P* < 0.0001, Fig 5A and 5B).

Differentiation of SCs was measured at 0, 24, 48, and 72 h following knockdown of mTOR at the beginning of differentiation and normal proliferation of the SCs for 72 h at 38° or 43°C (Table 5). At 0 h of differentiation, knockdown of *mTOR* showed no significant effect (*P* ≥ 0.1598) on SC differentiation in either the RBC2 or NC line compared to the control group at either 38 or 43°C. At 24 h of differentiation, SC differentiation was reduced 1.04-fold (*P* = 0.0046) in the NC *mTOR* knockdown group compared to the control group at 38°C, but no significant change was observed in the RBC2 line (*P* = 0.5897). At 43°C, differentiation of the SCs had a 1.05-fold (*P* = 0.0021) and 1.08-fold (*P* < 0.0001) reduction in the RBC2 and NC *mTOR* knockdown groups compared to control at 24 h. At 48 and 72 h of differentiation, SC differentiation decreased (*P* < 0.0001) in both the RBC2 and NC *mTOR* knockdown groups compared to the control groups at both 38° and 43°C. From 0 to 48 h, SC differentiation in both lines showed a linear increase in both the control and the *mTOR* knockdown groups at both 38° and 43°C. When *mTOR* was knocked down, the slope of the linear regression decreased 1.43-fold (*P* < 0.0001) and 1.09-fold (*P* = 0.0009) in the RBC2 and NC lines at 38°C from 0 to 48 h. At 43°C, the linear regression slope of the RBC2 and NC *mTOR* knockdown groups decreased 1.19-fold (*P* < 0.0001) and 1.19-fold (*P* < 0.0001) from 0 to 48 h. Significant interaction was observed among the effects of temperature, line, and knockdown of *mTOR* at 72 h (*P* < 0.0001).

**Table 5:** Effect of heat stress (43°C) and knockdown of *mTOR* at the beginning of differentiation on differentiation of RBC2 and NC line SCs^1^.

Line	Temperature^2^	Knockdown^3^	Sampling time				*P*-value^4^
			0 h	24 h	48 h	72 h	
RBC2	38	Control	6.93^c^^,^^w^ ± 0.36	15.79^f^^,^^x^ ± 0.43	40.45^f^^,^^y^ ± 1.08	51.77^g^^,^^z^ ± 0.59	< 0.0001
		mTOR	6.81^c^^,^^w^ ± 0.32	15.46^f^^,^^x^ ± 0.43	30.05^g^^,^^y^ ± 1.08	38.37^h^^,^^z^ ± 0.77	< 0.0001
	43	Control	12.31^b^^,^^w^ ± 0.32	51.54^d^^,^^x^ ± 0.47	72.35^d^^,^^z^ ± 0.97	68.15^e^^,^^y^ ± 0.54	< 0.0001
		mTOR	11.63^b^^,^^w^ ± 0.30	48.93^e^^,^^x^ ± 0.61	62.08^e^^,^^z^ ± 0.88	56.17^f^^,^^y^ ± 0.59	< 0.0001
NC	38	Control	12.36^b^^,^^w^ ± 0.36	67.98^b^^,^^x^ ± 0.61	111.20^b^^,^^z^ ± 1.08	83.60^b^^,^^y^ ± 0.59	< 0.0001
		mTOR	12.38^b^^,^^w^ ± 0.31	65.35^c^^,^^x^ ± 0.61	101.31^c^^,^^z^ ± 1.25	80.67^c^^,^^y^ ± 0.66	< 0.0001
	43	Control	24.75^a^^,^^w^ ± 0.36	75.19^a^^,^^x^ ± 0.47	118.00^a^^,^^z^ ± 2.20	99.95^a^^,^^y^ ± 0.66	< 0.0001
		mTOR	24.82^a^^,^^w^ ± 0.36	69.43^b^^,^^x^ ± 0.47	104.92^c^^,^^z^ ± 1.32	77.61^d^^,^^y^ ± 0.66	< 0.0001
*P*-value^5^		L	< 0.0001	< 0.0001	< 0.0001	< 0.0001	
		T	< 0.0001	< 0.0001	< 0.0001	< 0.0001	
		K	0.4613	< 0.0001	< 0.0001	< 0.0001	
		L × T × K	0.5239	0.5720	0.3674	< 0.0001	

^1^ Each value represents mean creatine kinase activity (Unit / well) ± Standard error of mean (SEM)

^2^ Incubation temperature (°C) during proliferation and differentiation

^3^ Control = transfecting cells with a negative control sequence; mTOR = knockdown of *mTOR*

^4^ Effect of sampling times within each cell line, temperature, and knockdown of *mTOR*

^5^ Effect of line (L), temperature (T), and knockdown of *mTOR* (K), and the interaction effect among line, temperature, and knockdown of *mTOR* (L × T × K) within each sampling time

^a-f^ Mean creatine kinase activity (Unit / well ± SEM) within a column (sampling time) without a common letter are significantly different

^w-z^ Mean creatine kinase activity (Unit / well ± SEM) within a row (temperature and cell line) without a common letter are significantly different

*P* ≤ 0.05 was considered as significant different

**Fig 6:**
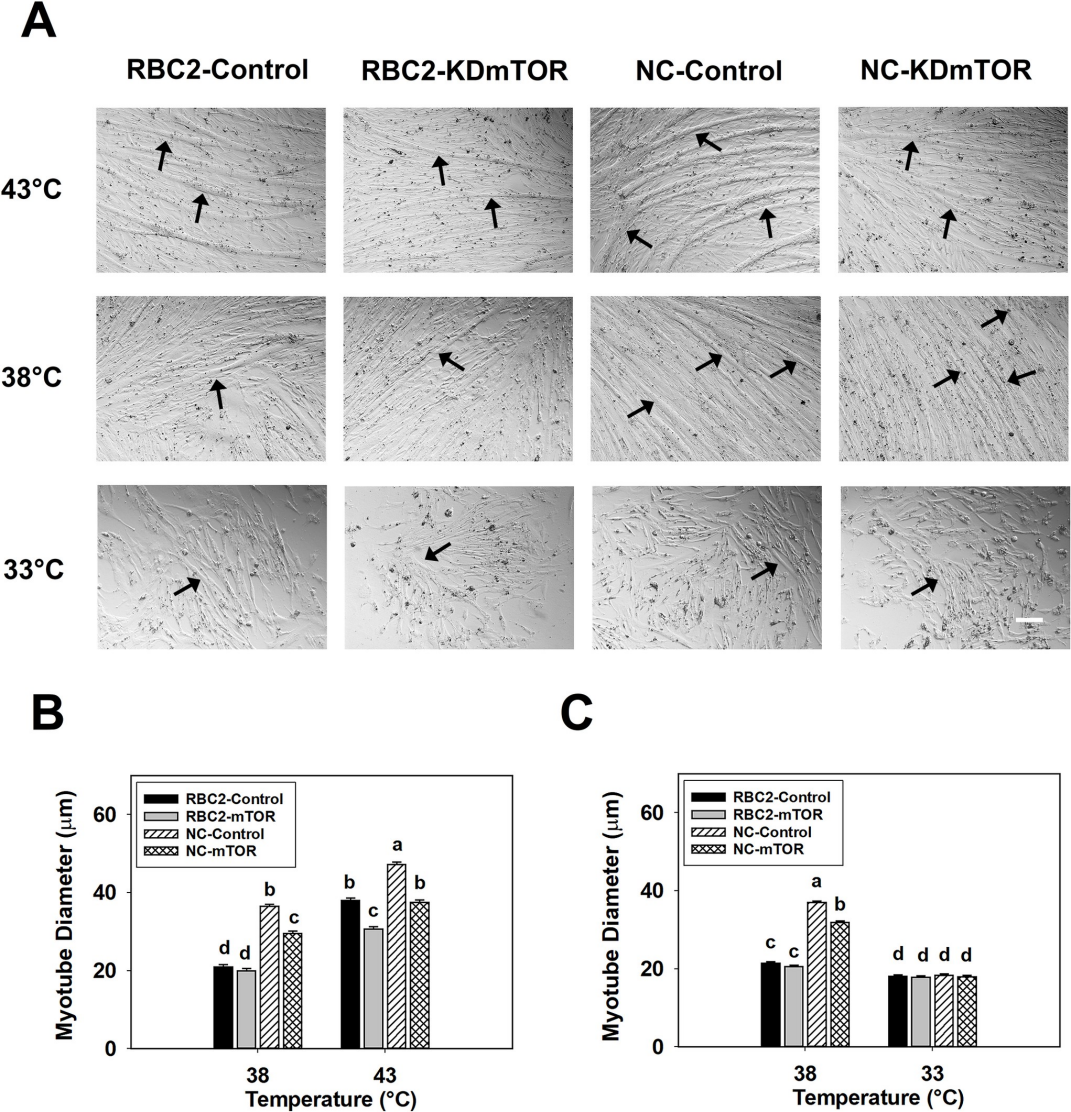
Effect of heat or cold thermal stress and siRNA-mediated knockdown of *mTOR* at the beginning of differentiation on the diameter of myotubes formed by RBC2 and NC line SCs. (A) Satellite cells from the RBC2 and NC lines proliferated normally at 38, 43, or 33°C and transfected with either a negative control siRNA (Control) or a siRNA targeting *mTOR* (KD mTOR) at 72 h of proliferation; photomicrographs were taken at 48 h of differentiation. (B) Diameter of myotubes in the RBC2 and NC lines at 38 or 43°C as shown in (A). (C) Diameter of myotubes in the RBC2 and NC lines at 38 or 33°C as shown in (A). Each graph bar represents a mean diameter of the myotubes, and each error bar represents a standard error of the mean value. Mean values with different letters are significantly different (*P* ≤ 0.05). Black arrows highlight the representative myotubes. Scale bar (White) = 100 μm.

Myotube diameter decreased only in the NC *mTOR* knockdown group (1.23-fold, *P* < 0.0001) at 38°C at 48 h of differentiation (Fig 6A and 6B). At 43°C, knockdown of *mTOR* decreased the diameter of myotubes in RBC2 and NC lines by 1.24-fold (*P* < 0.0001) and 1.26-fold (*P* < 0.0001) at 48 h of differentiation (Fig 6A and 6B). An interaction effect among the effects of temperature, line, and knockdown of *mTOR* was significant at 48 h of differentiation (*P* = 0.0318, Fig 6A and 6B).

#### 3.5.2 Effect of cold stress

After *mTOR* knockdown at the beginning of proliferation, differentiation was measured at 0, 24, 48, and 72 h of differentiation (Table 6). At each sampling time, SC differentiation significantly decreased (*P* < 0.0001) in the RBC2 and NC *mTOR* knockdown groups compared to the control groups at 38°C. At 33°C, decreased differentiation was observed in both the RBC2 and NC *mTOR* knockdown groups compared to the control groups at 0 h (*P* < 0.0001) and 48 h (*P* ≤ 0.0316). A greater fold change in SC differentiation was always observed in the RBC2 line SCs compared to the NC line at both 38° and 33°C at all sampling times. From 0 to 48 h, differentiation of the SCs in both lines showed a linear increase in both the control and *mTOR* knockdown groups at 38°C. The slope of the linear regression decreased 8.06-fold (*P* < 0.0001) and 2.72-fold (*P* < 0.0001) in the RBC2 and NC *mTOR* knockdown groups at 38°C. At 33°C, SC differentiation decreased significantly (*P* < 0.0001) in both line SCs at 24 and 48 h compared to 0 h in both the control and *mTOR* knockdown groups. A significant interaction effect was observed among the temperature, line, and knockdown of *mTOR* effects at 24 h (*P* < 0.0001) and 72 h (*P* < 0.0001).

**Table 6:** Effect of cold stress (33°C) and knockdown of *mTOR* at the beginning of proliferation on differentiation of RBC2 and NC line SCs^1^.

Line	Temperature^2^	Knockdown^3^	Sampling time				*P*-value^4^
			0 h	24 h	48 h	72 h	
RBC2	38	Control	5.32^b^^,^^w^ ± 0.39	11.37^b^^,^^x^ ± 0.70	55.55^b^^,^^y^ ± 0.92	67.51^a^^,^^z^ ± 1.21	< 0.0001
		mTOR	2.37^de^^,^^x^ ± 0.33	1.68^cd^^,^^x^ ± 0.82	8.10^d^^,^^y^ ± 0.63	10.94^d^^,^^z^ ± 1.06	< 0.0001
	33	Control	4.05^c^^,^^z^ ± 0.30	1.88^cd^^,^^x^ ± 0.53	1.99^f^^,^^yx^ ± 0.42	2.13^f^^,^^y^ ± 0.85	< 0.0001
		mTOR	1.84^e^^,^^z^ ± 0.25	0.49^d^^,^^y^ ± 0.57	0.28^g^^,^^y^ ± 0.63	0.49^f^^,^^y^ ± 0.85	< 0.0001
NC	38	Control	6.99^a^^,^^w^ ± 0.39	34.63^a^^,^^x^ ± 0.63	83.44^a^^,^^z^ ± 0.92	58.82^b^^,^^y^ ± 1.21	< 0.0001
		mTOR	4.49^bc^^,^^w^ ± 0.28	9.52^b^^,^^y^ ± 0.63	34.73^c^^,^^z^ ± 0.76	22.60^c^^,^^y^ ± 1.04	< 0.0001
	33	Control	4.94^b^^,^^y^ ± 0.30	3.18^c^^,^^x^ ± 0.63	3.61^e^^,^^yx^ ± 0.52	6.95^e^^,^^z^ ± 1.04	< 0.0001
		mTOR	2.94^d^^,^^z^ ± 0.27	1.72^cd^^,^^y^ ± 0.63	1.70^fg^^,^^y^ ± 0.48	2.71^f^^,^^z^ ± 0.85	< 0.0001
*P*-value^5^		L	< 0.0001	< 0.0001	< 0.0001	0.0018	
		T	< 0.0001	< 0.0001	< 0.0001	< 0.0001	
		K	< 0.0001	< 0.0001	< 0.0001	< 0.0001	
		L × T × K	0.7855	< 0.0001	0.5765	< 0.0001	

^1^ Each value represents mean creatine kinase activity (Unit / well) ± Standard error of mean (SEM)

^2^ Incubation temperature (°C) during proliferation and differentiation

^3^ Control = transfecting cells with a negative control sequence; mTOR = knockdown of *mTOR*

^4^ Effect of sampling times within each cell line, temperature, and knockdown of *mTOR*

^5^ Effect of line (L), temperature (T), and knockdown of *mTOR* (K), and the interaction effect among line, temperature, and knockdown of *mTOR* (L × T × K) within each sampling time

^a-f^ Mean creatine kinase activity (Unit / well ± SEM) within a column (sampling time) without a common letter are significantly different

^w-z^ Mean creatine kinase activity (Unit / well ± SEM) within a row (temperature and cell line) without a common letter are significantly different

*P* ≤ 0.05 was considered as significant different

Myotube diameter decreased 1.43-fold (*P* < 0.0001) and 1.99-fold (*P* < 0.0001) in the RBC2 and NC *mTOR* knockdown groups compared to the control groups at 38°C at 48 h of differentiation (Fig 5A and 5C). At 33°C, myotube diameter in the RBC2 and NC *mTOR* knockdown groups had a 1.06-fold (*P* = 0.0215) and 1.08-fold (*P* = 0.0028) reduction at 48 h of differentiation (Fig 5A and 5C). Significant interaction was observed among the effects of temperature, line, and knockdown of *mTOR* at 48 h of differentiation (*P* < 0.0001, Fig 5A and 5C).

When *mTOR* was knocked down at the beginning of differentiation and the SCs proliferated normally for 72 h at 38° or 33°C, differentiation was measured at 0, 24, 48, and 72 h of differentiation (Table 7). At 0 h of differentiation, no significant effect (*P* ≥ 0.1627) on SC differentiation was observed in either RBC2 or NC *mTOR* knockdown group compared to the control group at either 38 or 33°C. At 24 h of differentiation at the control temperature (38°C), SC differentiation showed a 1.06-fold (*P* < 0.0001) decrease only in the NC *mTOR* knockdown group compared to the control group. At 48 h of differentiation, a 1.37-fold (*P* < 0.0001) and 1.14-fold (*P* < 0.0001) reduction in SC differentiation in both the RBC2 and NC *mTOR* knockdown groups was observed only at 38°C compared to the control groups. Finally, at 72 h of differentiation, differentiation of SCs at 38°C was decreased 1.45-fold (*P* < 0.0001) and 1.09-fold (*P* < 0.0001) in the RBC2 and NC *mTOR* knockdown groups compared to the control groups. Under cold stress (33°C), knockdown of *mTOR* had no significant effect (*P* ≥ 0.3496) on SC differentiation in either the RBC2 or NC line at 24 or 48 h of differentiation. However, a significant reduction (1.29-fold, *P* = 0.0479) in SC differentiation was observed in the RBC2 *mTOR* knockdown group at 72 h. From 0 to 48 h, both lines showed a linear increase (*P* < 0.0001) in SC differentiation in both the control and *mTOR* knockdown groups at 38°C. When *mTOR* was knocked down at 38°C, the slope of the linear regression for SC differentiation was decreased 1.31-fold (*P* < 0.0001) and 1.15-fold (*P* < 0.0001) in the RBC2 and NC lines. At 33°C, SC differentiation decreased significantly (*P* < 0.0001) in the RBC2 line at 24, 48, and 72 h compared to 0 h in both the control and *mTOR* knockdown groups. In the NC line, differentiation decreased significantly (*P* < 0.0001) in both the control group and the *mTOR* knockdown group only at 24 h compared to 0 h at 33°C. Significant interaction was observed among the effects of temperature, line, and knockdown of *mTOR* at 24 h (*P* = 0.0080) and 72 h (*P* < 0.0001).

**Table 7:** Effect of cold stress (33°C) and knockdown of *mTOR* at the beginning of differentiation on differentiation of RBC2 and NC line SCs^1^.

Line	Temperature^2^	Knockdown^3^	Sampling time				*P*-value^4^
			0 h	24 h	48 h	72 h	
RBC2	38	Control	6.64^b^^,^^w^ ± 0.16	16.06^c^^,^^x^ ± 0.29	46.39^c^^,^^y^ ± 0.36	58.73^c^^,^^z^ ± 0.30	< 0.0001
		mTOR	6.36^b^^,^^w^ ± 0.21	15.56^c^^,^^x^ ± 0.41	33.95^d^^,^^y^ ± 0.29	40.42^d^^,^^z^ ± 0.30	< 0.0001
	33	Control	4.98^cd^^,^^z^ ± 0.21	2.65^e^^,^^w^ ± 0.29	3.55^e^^,^^x^ ± 0.29	4.57^f^^,^^y^ ± 0.37	< 0.0001
		mTOR	4.57^d^^,^^z^ ± 0.21	2.45^e^^,^^x^ ± 0.29	3.80^e^^,^^y^ ± 0.36	3.53^g^^,^^y^ ± 0.30	< 0.0001
NC	38	Control	12.64^a^^,^^w^ ± 0.25	64.52^a^^,^^x^ ± 0.41	117.32^a^^,^^z^ ± 0.36	85.26^a^^,^^y^ ± 0.30	< 0.0001
		mTOR	12.35^a^^,^^w^ ± 0.21	61.07^b^^,^^x^ ± 0.41	102.84^b^^,^^z^ ± 0.36	78.42^b^^,^^y^ ± 0.37	< 0.0001
	33	Control	5.40^c^^,^^y^ ± 0.21	4.78^d^^,^^x^ ± 0.33	4.35^e^^,^^x^ ± 0.29	9.35^e^^,^^z^ ± 0.30	< 0.0001
		mTOR	5.58^c^^,^^y^ ± 0.21	4.50^d^^,^^x^ ± 0.26	3.90^e^^,^^x^ ± 0.36	9.48^e^^,^^z^ ± 0.30	< 0.0001
*P*-value^5^		L	< 0.0001	< 0.0001	< 0.0001	< 0.0001	
		T	< 0.0001	< 0.0001	< 0.0001	< 0.0001	
		K	0.1857	0.0002	< 0.0001	< 0.0001	
		L × T × K	0.2990	0.0080	0.1827	< 0.0001	

^1^ Each value represents mean creatine kinase activity (Unit / well) ± Standard error of mean (SEM)

^2^ Incubation temperature (°C) during proliferation and differentiation

^3^ Control = transfecting cells with a negative control sequence; mTOR = knockdown of *mTOR*

^4^ Effect of sampling times within each cell line, temperature, and knockdown of *mTOR*

^5^ Effect of line (L), temperature (T), and knockdown of *mTOR* (K), and the interaction effect among line, temperature, and knockdown of *mTOR* (L × T × K) within each sampling time

^a-f^ Mean creatine kinase activity (Unit / well ± SEM) within a column (sampling time) without a common letter are significantly different

^w-z^ Mean creatine kinase activity (Unit / well ± SEM) within a row (temperature and cell line) without a common letter are significantly different

*P* ≤ 0.05 was considered as significant different

A significant decrease in myotube diameter was observed only in the NC *mTOR* knockdown group (1.16-fold, *P* < 0.0001) compared to the control group at 38°C at 48 h of differentiation (Fig 6A and 6C). However, at 33°C, knockdown of *mTOR* showed no significant effect (*P* > 0.4833) on myotube diameter in either the RBC2 or NC line (Fig 6A and 6C). Interaction effect among the effects of temperature, line, and knockdown of *mTOR* was significant at 48 h of differentiation (*P* < 0.0001, Fig 6A and 6C).

3.6. Effect of mTOR knockdown, thermal stress, and growth selection on myogenic transcriptional factor expression

#### 3.6.1 Effect of heat stress

**Fig 7:**
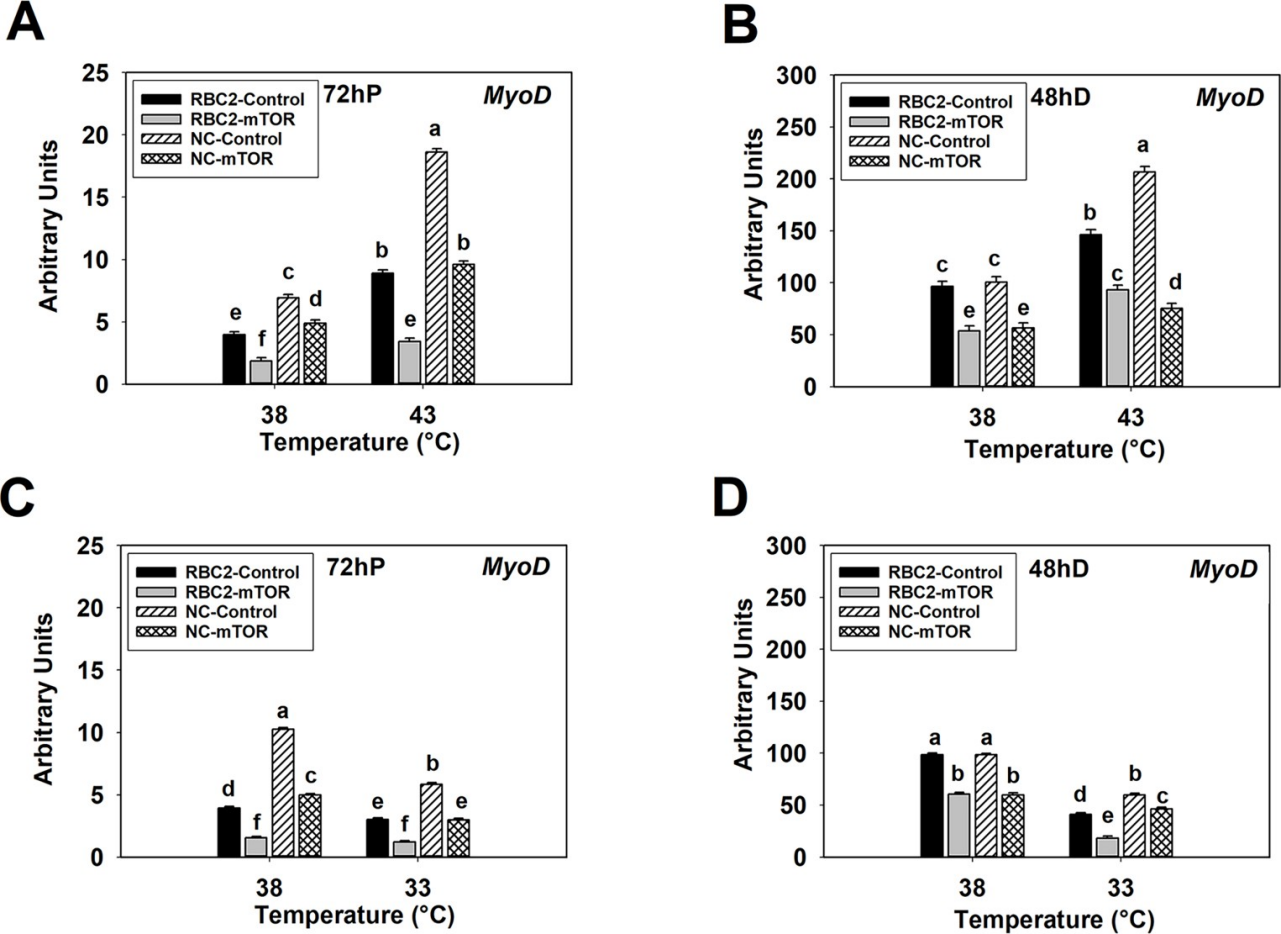
Effect of heat or cold thermal stress and siRNA-mediated knockdown of *mTOR* at the beginning of proliferation on the expression of *MyoD* in RBC2 and NC line SCs. After transfection with either a negative control siRNA (Control) or a siRNA targeting *mTOR* (mTOR), SCs proliferated at 38° or 43°C for 72 h (A) followed by 48 h of differentiation (B). After transfection with either a negative control siRNA (Control) or a siRNA targeting *mTOR* (mTOR), SCs proliferated at 38° or 33°C for 72 h (C) followed by 48 h of differentiation (D). Each graph bar represents a mean arbitrary unit, and each error bar represents a standard error of the mean. Mean values without a same letter are significantly different (*P* ≤ 0.05).

At 72 h of proliferation at 38°C, expression of *MyoD* showed a 2.14-fold (*P* < 0.0001) and 1.42-fold (*P* < 0.0001) reduction in the RBC2 and NC *mTOR* knockdown groups compared to the control groups (Fig 7A). At 43°C, *MyoD* expression decreased 2.59-fold (*P* < 0.0001) and 1.94-fold (*P* < 0.0001) in the RBC2 and NC *mTOR* knockdown groups (Fig 7A). At 48 h of differentiation, *MyoD* expression was lower (*P* < 0.0001) in the RBC2 and NC *mTOR* knockdown group SCs compared to the control groups at 38° and 43°C (Fig 7B). An interaction effect was significant among the temperature, line, and knockdown of *mTOR* effects at 72 h of proliferation (*P* < 0.0001) and 48 h of differentiation (*P* < 0.0001, Fig 7B).

**Fig 8:**
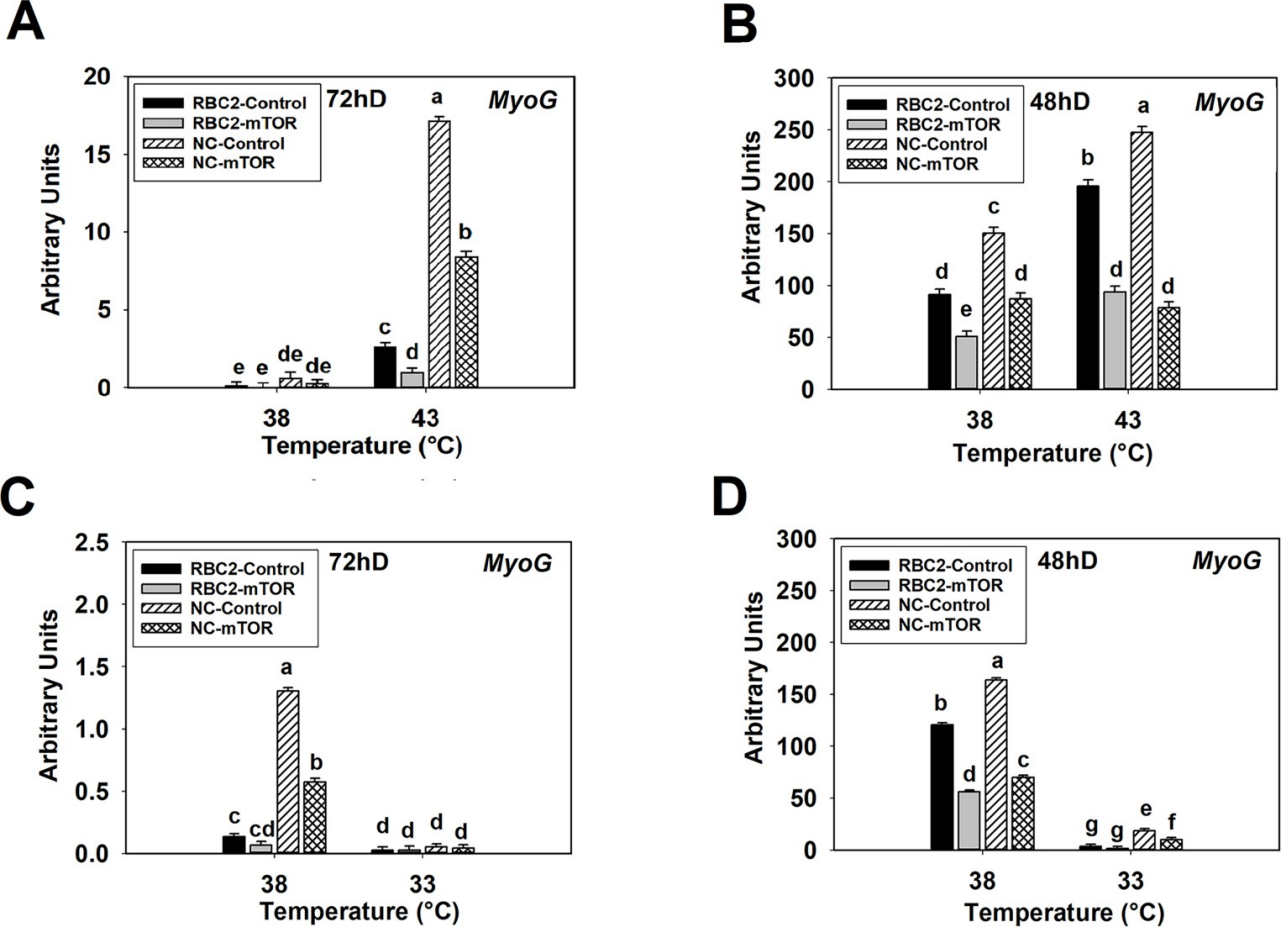
Effect of heat or cold thermal stress and siRNA-mediated knockdown of *mTOR* at the beginning of proliferation on the expression of *MyoG* in RBC2 and NC line SCs. After transfection with either a negative control siRNA (Control) or a siRNA targeting *mTOR* (mTOR), SCs proliferated at 38° or 43°C for 72 h (A) followed by 48 h of differentiation (B). After transfection with either a negative control siRNA (Control) or a siRNA targeting *mTOR* (mTOR), SCs proliferated at 38° or 33°C for 72 h (C) followed by 48 h of differentiation (D). Each graph bar represents a mean arbitrary unit, and each error bar represents a standard error of the mean. Mean values different letter are significantly different (*P* ≤ 0.05).

At 72 h of proliferation at the control temperature (38°C), *MyoG* expression showed no significant change (*P* ≥ 0.4386) in either the RBC2 or NC *mTOR* knockdown groups compared to the control groups (Fig 8A). Similarly, at 48 h of differentiation, expression of *MyoG* was 1.78-fold (*P* < 0.0001) and 1.72-fold (*P* < 0.0001) lower in the RBC2 and NC *mTOR* knockdown groups (Fig 8B). At 72 h of proliferation under heat stress (43°C), *MyoG* expression decreased 2.64-fold (*P* = 0.0005) and 2.03-fold (*P* < 0.0001) in the RBC2 and NC *mTOR* knockdown groups (Fig 8A). Similarly, *MyoG* decreased 2.09-fold (*P* < 0.0001) and 3.14-fold (*P* < 0.0001) in the RBC2 and NC *mTOR* knockdown groups at 48 h differentiation (Fig 8B). A significant interaction effect was observed among temperature, line, and knockdown of *mTOR* at 72 h of proliferation (*P* < 0.0001) and 48 h of differentiation (*P* = 0.0091, Fig 8B).

#### 3.6.2 Effect of cold stress

At 72 h of proliferation at 38°C, knockdown of *mTOR* decreased the expression of *MyoD* with a 2.60-fold (*P* < 0.0001) and 2.06-fold (*P* < 0.0001) reduction observed in the RBC2 and NC line SCs compared to the control groups (Fig 7C). Similarly, *MyoD* expression at 33°C decreased 1.63-fold (*P* < 0.0001) and 1.96-fold (*P* < 0.0001) in the RBC2 and NC *mTOR* knockdown groups (Fig 7C). After 48 h of differentiation at 38°C, a significant 5.39-fold (*P* < 0.0001) and 1.64-fold (*P* < 0.0001) reduction in *MyoD* expression was observed in the RBC2 and NC *mTOR* knockdown groups compared to the control groups, and *MyoD* expression was also reduced under cold treatment [a 2.24-fold (*P* < 0.0001) and 1.29-fold (*P* < 0.0001) decrease in RBC2 and NC *mTOR* knockdown groups, respectively] (Fig 7D). There was a significant interaction among the effects of temperature, line, and knockdown of *mTOR* at 72 h of proliferation (*P* < 0.0001) and 48 h of differentiation (*P* = 0.0385, Fig 7D).

Expression of *MyoG* at 72 h of proliferation at the control temperature was decreased 2.27-fold (*P* < 0.0001) only in the NC *mTOR* knockdown group compared to the control (Fig 8C). No significant effect on *MyoG* expression was observed at 33°C (*P* ≥ 0.7783, Fig 8C). At 48 h of differentiation at 38°C, *MyoG* expression reduced 2.15-fold (*P* < 0.0001) and 2.34-fold (*P* < 0.0001) in the RBC2 and NC *mTOR* knockdown group SCs (Fig 8D). Cold stress (33°C) reduced expression of *MyoG* only in the NC *mTOR* knockdown group (*P* = 0.0050, Fig 8D). There was a significant interaction among the effects of temperature, line, and knockdown of *mTOR* at 72 h of proliferation (*P* < 0.0001) and 48 h of differentiation (*P* = 0.0002, Fig 8D).

## 4. Discussion

Post-hatch thermal stress affects growth and structure of poultry p. major muscle [7–9, 21, 71], in part, through changes in the proliferation and differentiation of SCs [16, 17, 22, 60, 71]. Growth selection for fast-growing, heavy-weight birds is a primary factor affecting thermal stress-induced changes in SC activity [16, 22]. At the transcriptional level, expression of genes related to mTOR and S6K signal transduction are highly affected by thermal stress during turkey p. major muscle SC differentiation [25]. Furthermore, both hot [33] and cold [59] thermal stress can affect mTOR signal transduction in chicken skeletal muscle. Altered mTOR signal transduction affects protein synthesis in cooperation with a downstream effector protein, S6K [33, 53], and the amount of SC intracellular protein may determine the hypertrophic potential of the skeletal muscle. Thus, both heat and cold stress may affect the hypertrophic growth of the turkey breast muscle through regulation of mTOR/S6K signal transduction in SCs.

Satellite cells represent a heterogenous population of cells [72–77]. Thus, SCs from slow and fast muscles will express slow and fast contractile protein isoforms similar to the myofibers they are derived from [76, 77], and can vary in proliferation and differentiation rates [78] and metabolic properties [79]. Even SCs isolated from the same myofiber can differ in proliferation and differentiation [68], growth factor responsiveness [68, 69], and expression of myogenic regulatory factors [80, 81]. Genetic selection for growth changes the properties of SCs in the p. major muscle, with birds selected for increased growth and breast muscling having elevated proliferation and differentiation rates and increased temperature sensitivity [16, 22].

With the intensive growth selection, modern faster-growing meat-type poultry lines have increased breast proportion [82], greater breast meat yield [82], and upregulated expression of growth-promoting genes in the p. major muscle [83] compared to the slower-growing historic lines. The enhanced growth characteristics of breast muscle may be associated with changes in mTOR/S6K signal transduction in p. major muscle SCs. Data from the current study found the modern NC line SCs had greater activity in mTOR/S6K signal transduction compared to the RBC2 line independent of temperature, suggesting SCs of the modern commercial line have a greater hypertrophic potential through protein synthesis than the RBC2 line. In support of increased hypertrophic potential, myotubes formed by the cultured NC line SCs had a significantly larger diameter than those formed by the RBC2 line. This is similar to results by Velleman et al. [19], in that the p. major muscle from growth selected F-line turkeys (selected only for increased 16-week body weight from the RBC2 line) had myofibers with larger diameters than the RBC2 line. Thus, growth selection may have increased hypertrophic potential of the p. major muscle in an mTOR/S6K-dependent mechanism. Since mTOR/S6K-dependent protein synthesis can determine the size of skeletal muscle in many species [36, 54–57], the increased mTOR/S6K activity may drive the formation of excessive hypertrophic myofibers (giant fibers) in the p. major muscle of faster-growing turkeys. Giant fibers decrease the space for connective tissue [19] and reduce capillary support [19–21, 84]. These changes in breast muscle morphology are associated with increasing oxidative stress [85], a primary cause of degenerative myopathies. Furthermore, being an anaerobic muscle [86, 87], breast muscle already has reduced capillary support compared to an oxidative muscle. Birds with higher growth rate have greater glycolytic potential to generate more anaerobic by-products such as lactic acid [88]. The reduced capillary supply in breast muscle will limit the removal lactic acid, which may further drive muscle degeneration.

Environmental thermal stress may further affect the hypertrophic potential of avian breast muscle. Both hot [33] and cold [59] thermal stress differentially affects signal transduction through the mTOR pathway in chicken skeletal muscle. In the current study, heat stress increased the activity of mTOR/S6K pathway and the diameter of myotubes in both lines. Similarly, wider myotubes were also observed in both the growth selected F-line and the RBC2 line SCs with the elevated temperatures [22]. Since the breast muscle of faster-growing birds has already showed impaired morphological structure under normal temperatures due to the formation of giant myofibers [19–21, 83], heat stress-induced enhanced activity in mTOR/S6K may exacerbate this condition. In support of this postulate, post-hatch heat stress was reported to further reduce the capillary density [21, 71] and increase muscle degeneration [21] in chicken p. major muscle. Furthermore, faster-growing birds with a higher metabolic rate generate more metabolic heat [89], that is harder to dissipate due to the reduced capillary density in the p. major muscle [2, 20, 21, 84]. Since birds are homeotherms and have limited ability to maintain the body temperature [1, 2], excessive metabolic heat may further amplify the effect of environmental heat stress.

With cold stress, both the activity of the mTOR/S6K pathway and myotube diameter decreased, with the NC line showing a greater reduction than the RBC2 line. Thus, cold stress during the period of maximal SC mitotic activity may suppress breast muscle mass accretion by reducing mTOR/S6K-mediated protein synthesis. However, in the chicken leg muscle, an aerobic muscle, increased mTOR activity was observed when newly hatched chickens were constantly challenged during the first week after hatch with chronic cold stress (5.3° to 12.3°C colder than control) [59]. Therefore, effect of cold stress on the activity of mTOR signal transduction may be dependent on muscle type and the intensity and duration of the cold stress.

Previous studies have shown both proliferation and differentiation in avian p. major muscle SCs are affected by thermal stress [16, 17, 22, 60]. Since mTOR signal transduction regulates the proliferation and differentiation of mammalian [26–30] and avian [31] SCs, changes in mTOR signal transduction may alter SC activity under thermal stress. In the present study, suppression of mTOR/S6K pathway activity by *mTOR* knockdown at the beginning of proliferation, resulted in both decreased SC proliferation and *MyoD* expression in both lines independent of temperature, with a greater reduction observed in the RBC2 line at 43°C. Although heat stress increased the proliferation of SCs in both lines, the NC line still exhibited a higher proliferation rate and *MyoD* expression than the RBC2 line. These data suggest that under heat stress the proliferation of p. major muscle SCs from the modern commercial line was less dependent on the mTOR/S6K signal transduction than the RBC2 line. It is possible that growth selection may have increased the activity of other alternative signaling pathways to regulate the heat stress-induced increase in SC proliferation. For example, Reed et al. [24] reported the expression of *Wnt7a* (wingless-type mouse mammary tumor virus integration site family, member 7a), which is important in promoting SC proliferation [90], was much higher in the proliferating F-line SCs compared to the RBC2 line under heat stress. With more alternative signaling pathways regulating SC proliferation, faster-growing poultry are more likely to maintain a larger SC pool under heat stress. Since the size of SC pool affects muscle mass accretion in chicken p. major muscle [7, 8], a larger SC pool induced by heat stress may enhance p. major muscle growth and mass accretion in faster-growing poultry.

**Fig 9:**
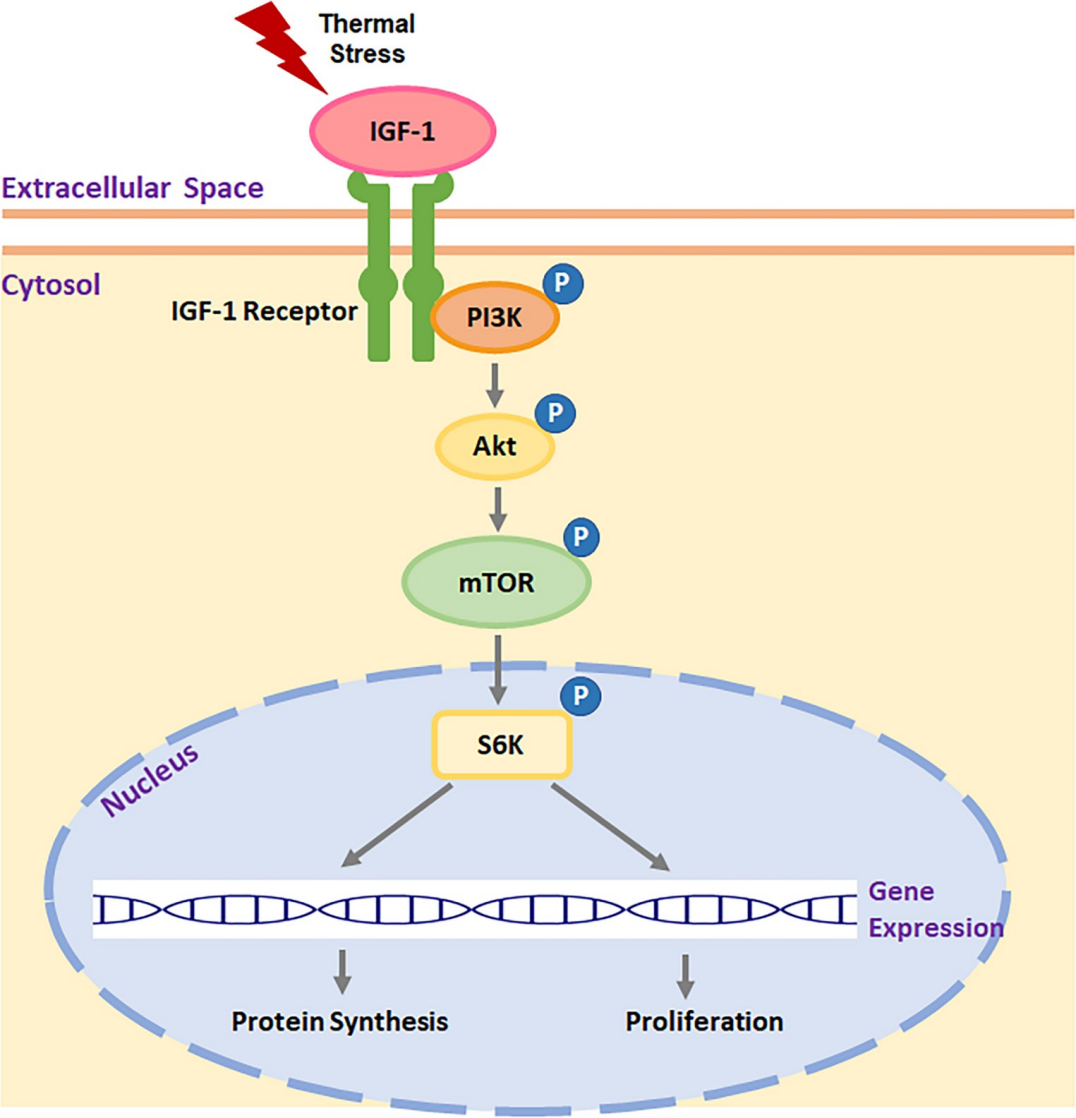
Schematic illustration of heat stress-induced IGF-1 regulating SC proliferation and protein synthesis through mTOR/S6K pathway. IGF-1: insulin-like growth facter-1, PI3K: phosphoinositide 3-kinase, Akt: protein kinase B, mTOR: mechanistic target of rapamycin, S6K: p70 S6 kinase.

Heat stress-induced increase in SC proliferation was accompanied by the increased expression of insulin-like growth facter-1 (IGF-1) in chicken p. major muscle [17]. Ma et al. [33] showed that heat stress affected chicken breast muscle mass through IGF-1/mTOR/S6K pathway. A schematic illustration of the heat stress-induced IGF-1/mTOR/S6K pathway is presented in Fig 9. As an upstream signal, IGF-1 can interact with IGF-1 receptor, activating mTOR/S6K signal transduction via PI3K/Akt pathway [38, 49, 91]. Previous studies have shown that IGF-1 can regulate myogenic cell proliferation [29, 92] and skeletal muscle hypertrophy [33, 38] through mTOR/S6K pathway in mammals and birds. Thus, IGF-1 may be one of the upstream factors that can respond to heat stress and induce SC proliferation and protein synthesis through mTOR/S6K signal transduction in avian p. major muscle SCs.

Under cold stress, SCs of the NC line showed a greater reduction in both proliferation and *MyoD* expression with *mTOR* knockdown compared to the RBC2 line. Both the cold stress and the inhibition of the mTOR/S6K pathway diminished the line difference in SC proliferation and *MyoD* expression. At the transcriptional level, a greater number of genes was altered by cold stress than by heat stress in proliferating SCs, particularly in the faster-growing F-line compared to the RBC2 line [24]. These results suggest SCs from faster-growing turkeys have a lower tolerance to cold stress in maintaining cell proliferation than SCs isolated from slower-growing turkeys. Thus, cold stress decreased the proliferation of the p. major muscle SCs, in part, by inhibiting the activity of mTOR/S6K pathway. Since the mitotic activity of SCs reaches a peak during the first week after hatch [14, 15], cold stress during this period will greatly reduce the size of the cell pool accumulated through the proliferation of SCs. As shown by previous *in vivo* studies [7, 8], thermal stress-induced reduction in SC pool limited long-term mass accretion of poultry breast muscle.

With regard to SC differentiation, signal transduction through mTOR has been shown to affect the myogenic differentiation of SCs in mouse skeletal muscle [26–28]. In turkey p. major muscle SCs, suppressing activity of the mTOR/S6K pathway by *mTOR* knockdown at the beginning of proliferation, inhibited myogenic differentiation, reduced myotube diameter, and decreased *MyoG* expression in both lines independent of temperature. With heat stress, differentiation of the NC line was less dependent on the mTOR/S6K pathway, but still showed a higher creatine kinase activity and *MyoG* expression compared to the RBC2 line even though *mTOR* was knocked down. Other signal pathways involved in stimulating myogenic differentiation may have been enhanced by growth selection, and therefore, maintaining SC differentiation at a higher level under heat stress. For example, expression of neuropeptide Y (*NPY*) [25, 93] and neuropeptide Y receptor-5 (*NPY5R*) [93] was upregulated by the heat stress to a greater extent in the growth-selected F-line SCs compared to the RBC2 line during differentiation. The activation of chicken NPY5R by NPY initiates mitogen-activated protein kinase/extracellular signal-regulated kinase (MAPK/ERK) pathway [94], which has been reported to promote both the proliferation and differentiation of myogenic SCs in humans [95]. Further studies are needed to assess if the MAPK/ERK pathway is involved in thermal stress-induced change in the proliferation or differentiation of avian p. major muscle SCs.

Expression of genes involved in mTOR and S6K signal transduction were greatly downregulated by cold stress, during the differentiation of p. major muscle SCs, independent of cell line [25]. Since the activity of mTOR/S6K pathway has already been suppressed to an extremely low level under the cold stress, few inhibitory effects on SC differentiation, myotube diameter, and *MyoG* expression were observed upon the knockdown of *mTOR*. Other than mTOR/S6K pathway, expression of myocyte enhancer factor 2C (*MEF2C*) was also greatly downregulated at cold temperatures in differentiating p. major muscle SCs [25]. As a transcriptional factor, MEF2C can regulate SC myogenesis in skeletal muscle in cooperation with multiple signal transductions including p38 MAPK [96] and calcium-dependent [97] pathways. Thus, results of the present study suggest cold stress can largely diminish the effect of growth selection on the myogenic differentiation of the SCs, in part, by suppressing the mTOR/S6K pathway.

Thermal stress-induced changes in SC differentiation are timing-dependent. In the present study, proliferating SCs were more dependent on mTOR/S6K signal transduction in the determination of myogenic differentiation and myotube diameter than differentiating SCs independent of temperature. Similarly, Xu et al. [16] reported that SCs during proliferation were more responsive to thermal stress in the determination of myogenic differentiation than during differentiation. Furthermore, SCs from both lines showed increased *MyoG* expression during proliferation, and this increase was interrupted by knockdown of *mTOR* at the beginning of proliferation. Taken together, these data suggest that thermal stress-induced change in myogenic potential was determined as early as the proliferation period, and this change may attribute to the altered activity of mTOR/S6K pathway. Since SCs exhibit their peak mitotic activity during the first week after hatch [14, 15], post-hatch thermal stress during this period may not only change the size of SC pool, but also greatly alter the myogenic potential of the p. major muscle SCs. With altered proliferation and differentiation, SC-mediated myofiber hypertrophy will be affected, altering development and growth of the p. major muscle.

In summary, selection for growth has increased the activity of mTOR/S6K pathway in the p. major muscle SCs, and this appears to be independent of temperature. Heat stress further elevated the activity of mTOR/S6K pathway, while cold stress showed an inhibitory effect independent of cell line (turkey type). Signal transduction through mTOR/S6K did affect thermal stress-induced changes in SC proliferation and differentiation in a growth-dependent manner. The proliferation and differentiation of the SCs from the faster-growing turkeys was less dependent on the mTOR/S6K pathway compared to the slower-growing turkeys under heat stress. In contrast, the faster-growing line SCs were more sensitive to the cold stress-induced reduction in mTOR/S6K pathway activity with their proliferation and differentiation showing a greater reduction than the slower-growing line. Furthermore, proliferating SCs were more dependent on mTOR/S6K-mediated myogenic differentiation compared to the differentiating SCs independent of temperature and cell line. Therefore, both heat and cold stress immediately after hatch can affect p. major muscle hypertrophic potential by changing the proliferation and differentiation of SCs, in part, through mTOR/S6K pathway in a growth-dependent manner. Increased proliferation and differentiation of the heat challenged-SCs may further promote the formation of giant myofibers, decreasing connective tissue spacing and capillary density, and resulting in degenerative damage to the turkey p. major muscle.

## Supporting information

S1 Fig Phosphorylation profiles of mTOR and S6K in RBC2 and NC line SCs. (A) Protein levels of the unphosphorylated and phosphorylated forms of mTOR and S6K, and an internal control β-actin in SCs from the RBC2 and NC lines cultured at 38°C was determined with western blot analysis at 72 h of proliferation (72hP) and 24 h (24hD), 48 h (48hD), and 72 h (72hD) of differentiation. (B) The densitometric ratio of phosphorylated to unphosphorylated mTOR as shown in (A) was analyzed at each sampling time for each treatment group. (C) The densitometric ratio of phosphorylated to unphosphorylated S6K as shown in (A) was analyzed at each sampling time for each treatment group. (D) Protein levels of the unphosphorylated and phosphorylated forms of mTOR and S6K, and an internal control β-actin in SCs from the RBC2 and NC lines cultured at 43°C was determined with western blot analysis. (E) The densitometric ratio of phosphorylated to unphosphorylated mTOR as shown in (D) was analyzed at each sampling time for each treatment group. (F) The densitometric ratio of phosphorylated to unphosphorylated S6K as shown in (D) was analyzed at each sampling time for each treatment group. (G) Protein levels of the unphosphorylated and phosphorylated forms of mTOR and S6K, and an internal control β-actin in SCs from the RBC2 and NC lines cultured at 33°C was determined with western blot analysis. (H) The densitometric ratio of phosphorylated to unphosphorylated mTOR as shown in (G) was analyzed at each sampling time for each treatment group. (I) The densitometric ratio of phosphorylated to unphosphorylated S6K as shown in (G) was analyzed at each sampling time for each treatment. Molecular weight and name of each target protein is shown on the left and right side of each figure, respectively in (A), (D), and (G). Each graph bar represents a mean ratio, and each error bar represents a standard error of the mean value. Mean values with different letter are significantly different (*P* ≤ 0.05).
(TIF)

S1 Raw images Original blot images. (PDF)
