# Supplementary figures and images for: Thermal stress affects proliferation and differentiation of turkey satellite cells through the mTOR/S6K pathway in a growth-dependent manner
Source: PLoS One. 2022 Jan 13;17(1):e0262576. doi: 10.1371/journal.pone.0262576 (PMC8758067; doi:10.1371/journal.pone.0262576)

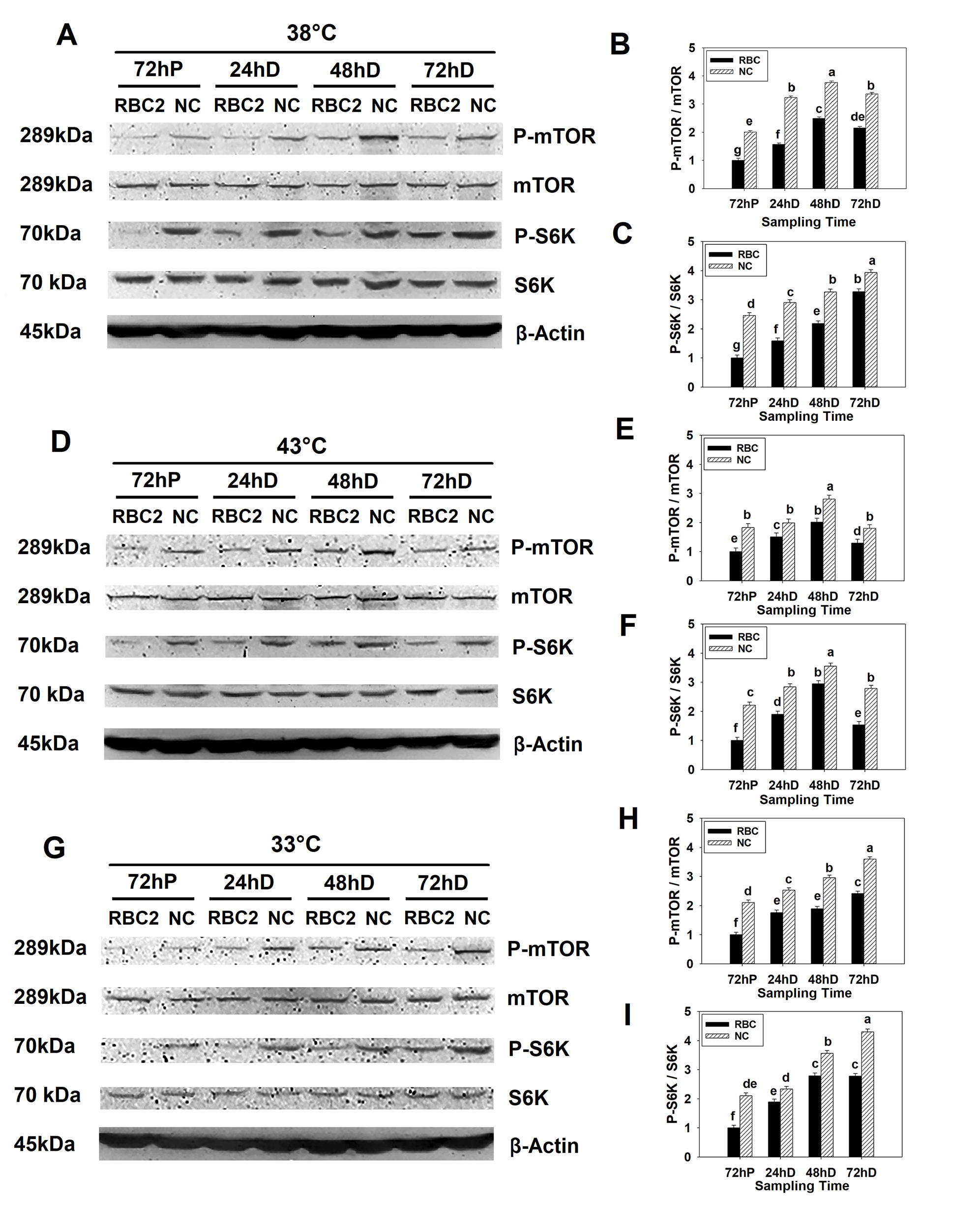

Supplement: S1 Fig — (A) Protein levels of the unphosphorylated and phosphorylated forms of mTOR and S6K, and an internal control β-actin in SCs from the RBC2 and NC lines cultured at 38°C was determined with western blot analysis at 72 h of proliferation (72hP) and 24 h (24hD), 48 h (48hD), and 72 h (72hD) of differentiation. (B) The densitometric ratio of phosphorylated to unphosphorylated mTOR as shown in (A) was analyzed at each sampling time for each treatment group. (C) The densitometric ratio of phosphorylated to unphosphorylated S6K as shown in (A) was analyzed at each sampling time for each treatment group. (D) Protein levels of the unphosphorylated and phosphorylated forms of mTOR and S6K, and an internal control β-actin in SCs from the RBC2 and NC lines cultured at 43°C was determined with western blot analysis. (E) The densitometric ratio of phosphorylated to unphosphorylated mTOR as shown in (D) was analyzed at each sampling time for each treatment group. (F) The densitometric ratio of phosphorylated to unphosphorylated S6K as shown in (D) was analyzed at each sampling time for each treatment group. (G) Protein levels of the unphosphorylated and phosphorylated forms of mTOR and S6K, and an internal control β-actin in SCs from the RBC2 and NC lines cultured at 33°C was determined with western blot analysis. (H) The densitometric ratio of phosphorylated to unphosphorylated mTOR as shown in (G) was analyzed at each sampling time for each treatment group. (I) The densitometric ratio of phosphorylated to unphosphorylated S6K as shown in (G) was analyzed at each sampling time for each treatment. Molecular weight and name of each target protein is shown on the left and right side of each figure, respectively in (A), (D), and (G). Each graph bar represents a mean ratio, and each error bar represents a standard error of the mean value. Mean values with different letter are significantly different (P ≤ 0.05). (TIF) [file pone.0262576.s001.tif]

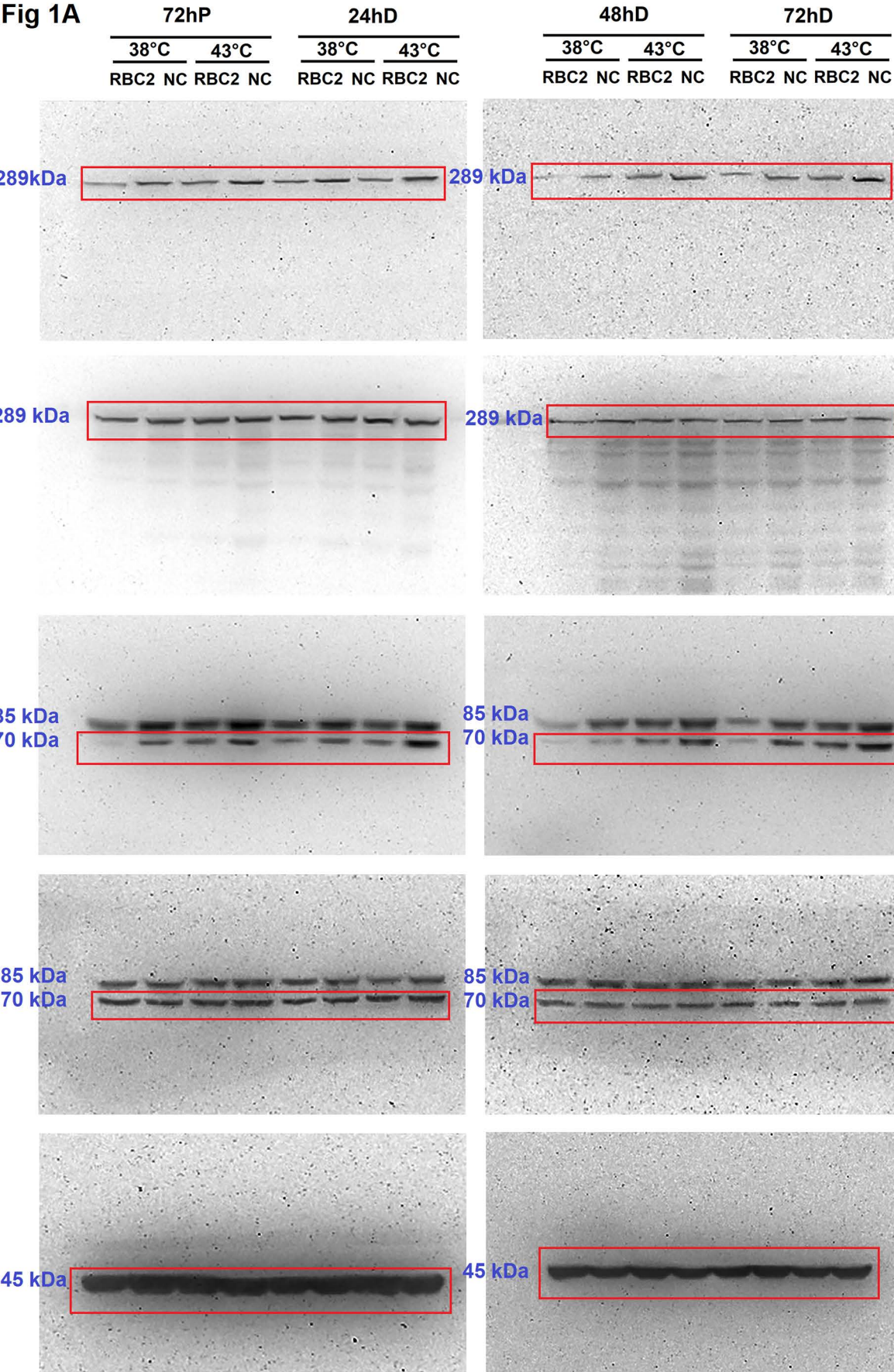

**Fig 2A**

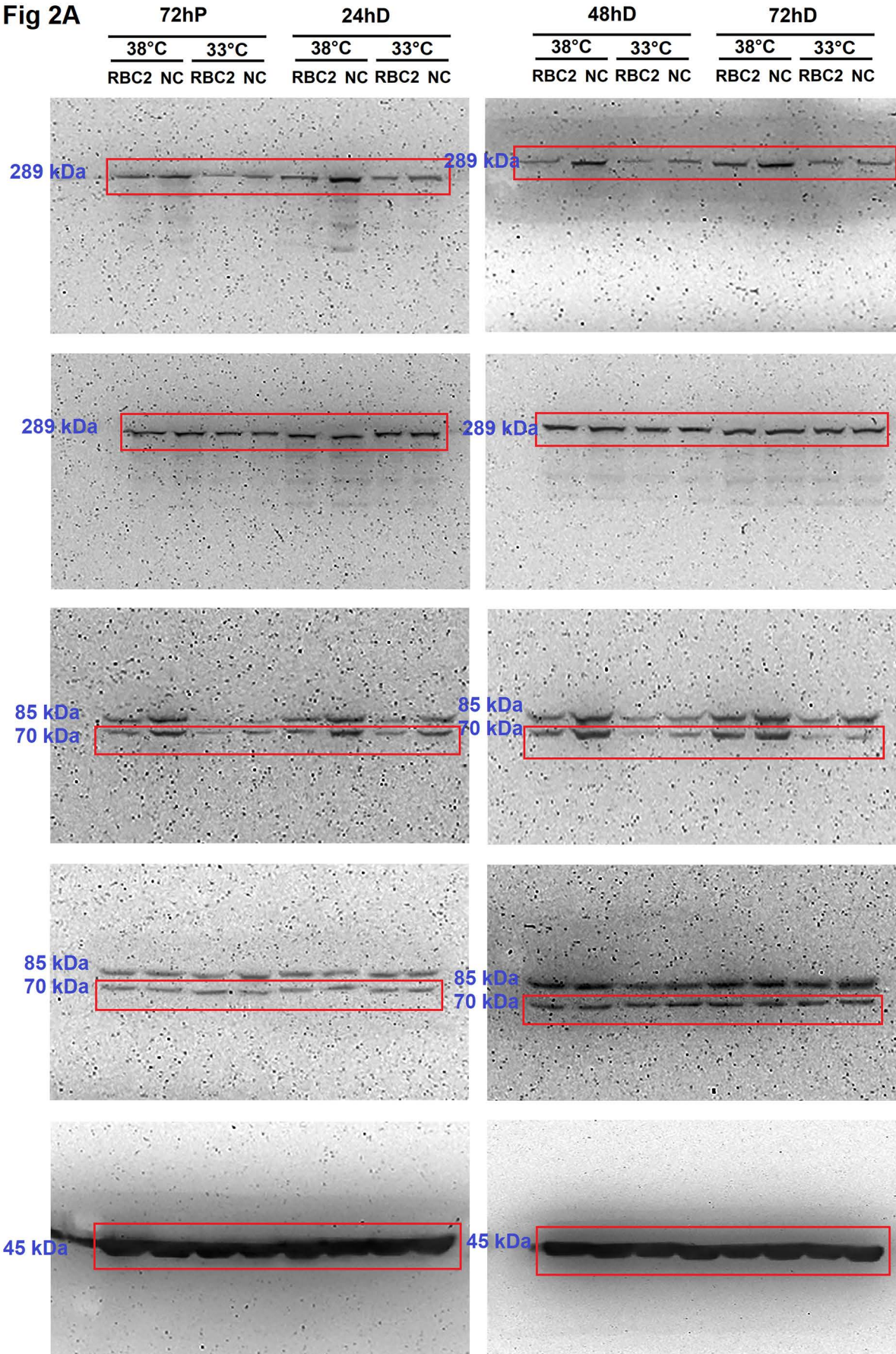

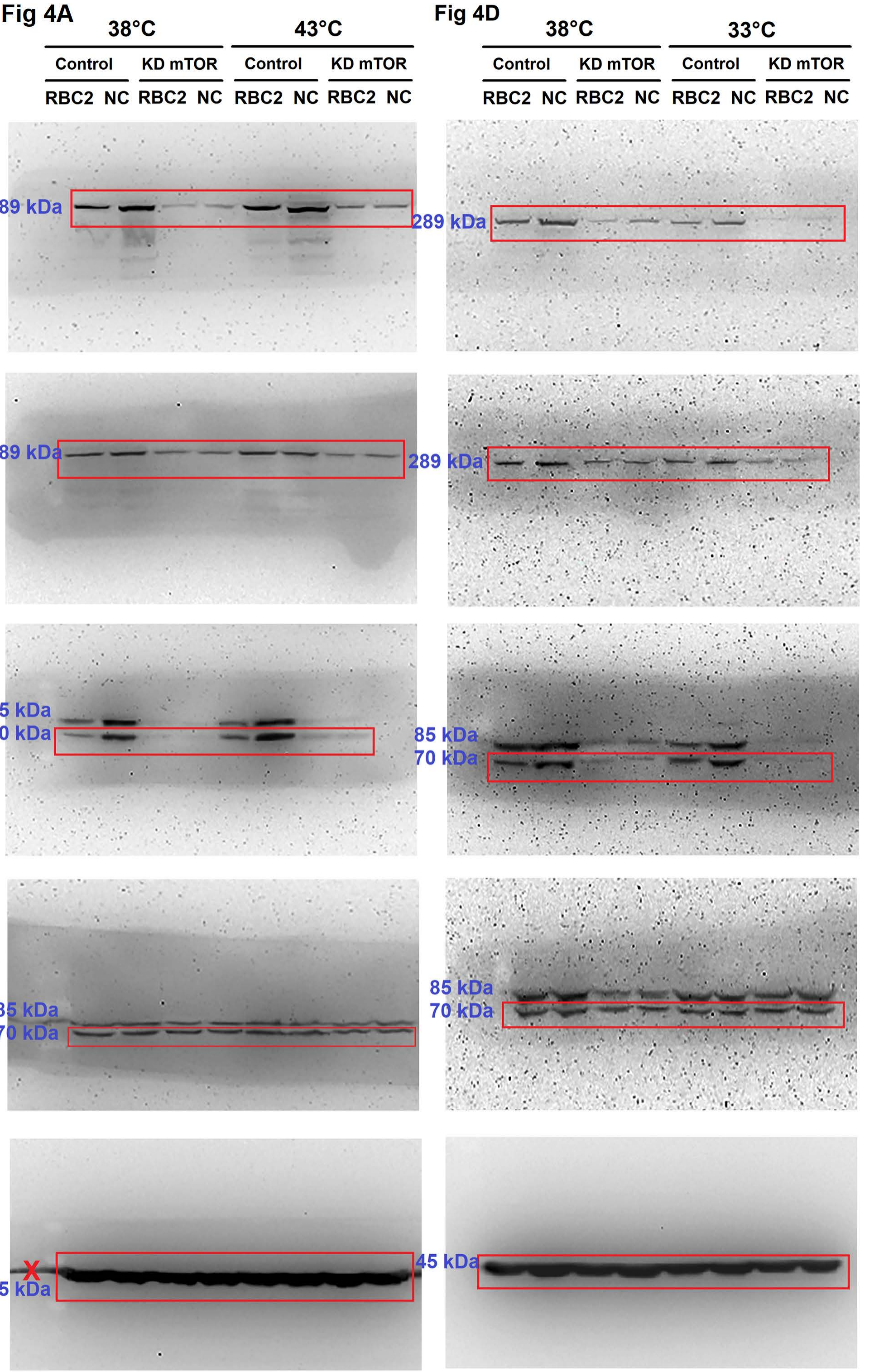

**S1 Fig A**

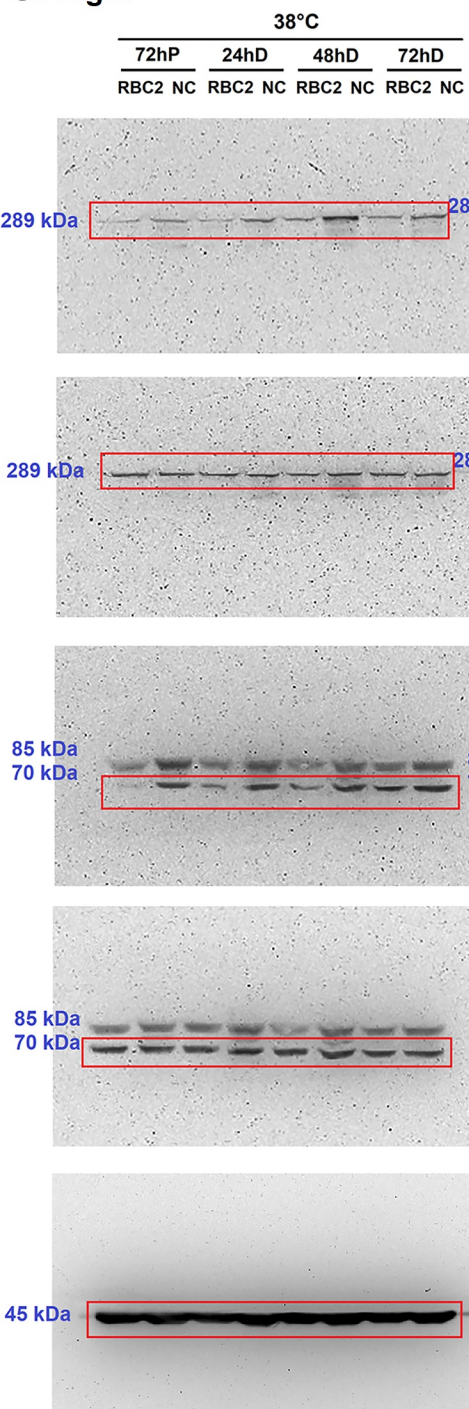

**S1 Fig D**

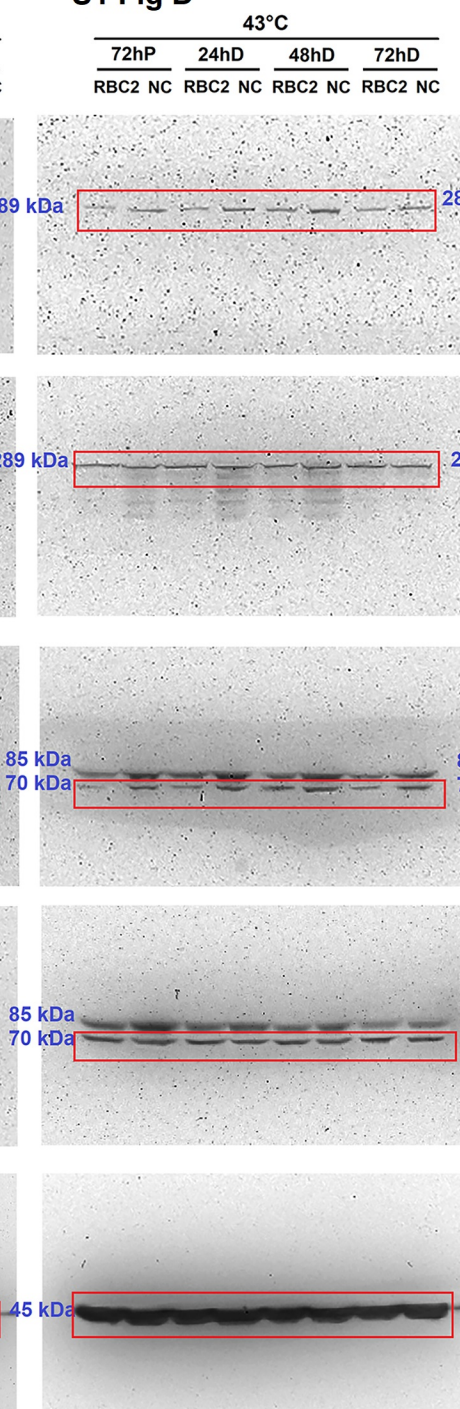

**S1 Fig G**

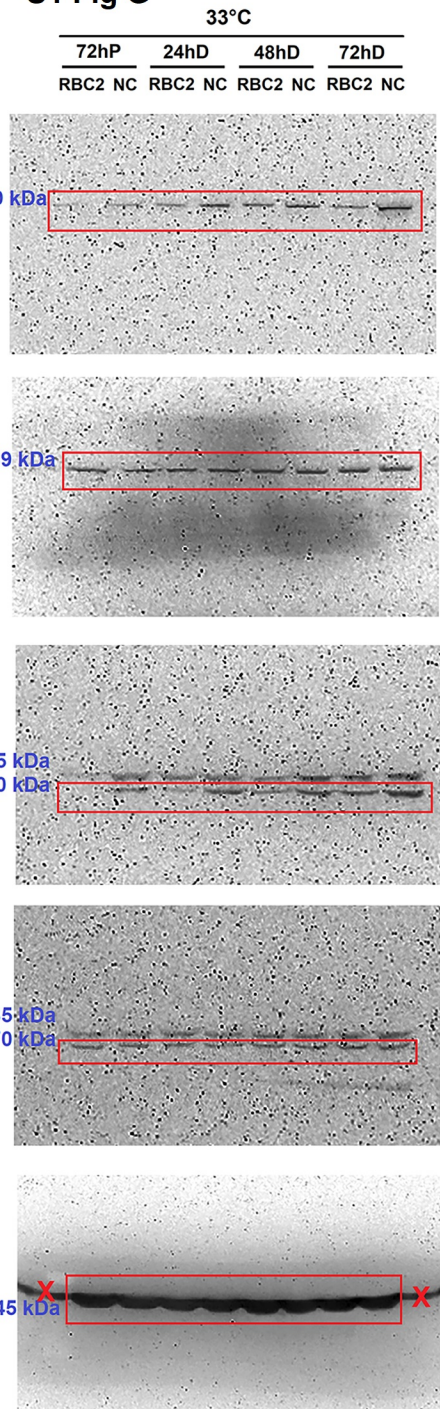

Supplement: S1 Raw images — (PDF) [file pone.0262576.s002.pdf]
